# Stirring the false vacuum via interacting quantized bubbles on a 5,564-qubit quantum annealer

**DOI:** 10.1038/s41567-024-02765-w

**Published:** 2025-02-04

**Authors:** Jaka Vodeb, Jean-Yves Desaules, Andrew Hallam, Andrea Rava, Gregor Humar, Dennis Willsch, Fengping Jin, Madita Willsch, Kristel Michielsen, Zlatko Papić

**Affiliations:** 1 Jülich Supercomputing Centre, Institute for Advanced Simulation, Forschungszentrum Jülich, Jülich, Germany; 2 Institute of Science and Technology Austria (ISTA), Klosterneuburg, Austria; 3 School of Physics and Astronomy, University of Leeds, Leeds, UK; 4 RWTH Aachen University, Aachen, Germany; 5 Department of Complex Matter, Jožef Stefan Institute, Ljubljana, Slovenia; 6 Department of Physics, Faculty for Mathematics and Physics, University of Ljubljana, Ljubljana, Slovenia; 7 Faculty of Medical Engineering and Technomathematics, University of Applied Sciences Aachen, Jülich, Germany; 8 AIDAS, Jülich, Germany

**Keywords:** Quantum simulation, Statistical physics

## Abstract

False vacuum decay—the transition from a metastable quantum state to a true vacuum state—plays an important role in quantum field theory and non-equilibrium phenomena such as phase transitions and dynamical metastability. The non-perturbative nature of false vacuum decay and the limited experimental access to this process make it challenging to study, leaving several open questions regarding how true vacuum bubbles form, move and interact. Here we observe quantized bubble formation in real time, a key feature of false vacuum decay dynamics, using a quantum annealer with 5,564 superconducting flux qubits. We develop an effective model that captures both initial bubble creation and subsequent interactions, and remains accurate under dissipation. The annealer reveals coherent scaling laws in the driven many-body dynamics for more than 1,000 intrinsic qubit time units. This work provides a method for investigating false vacuum dynamics of large quantum systems in quantum annealers.

## Abstract (web summary)

Bubble formation is a signal of false vacuum decay, in which a system transitions from a local energy minimum to a true vacuum. Now, simulations on a quantum annealer show how interactions between bubbles drive the long-time dynamics of this process.

## Main

Nearly half a century ago, Coleman proposed the idea that our Universe may have cooled down into a metastable ‘false vacuum’ state after the Big Bang, and the time of tunnelling to the ground state or ‘true vacuum’ was estimated to be comparable to the lifetime of the Universe^1^. The idea was then further applied to various cosmological scenarios^2–8^, with ongoing attempts to observe the signatures of false vacuum decay in gravitational waves^9^.

The dynamics of false vacuum decay are believed to consist of ‘bubbles’ of true vacuum forming in the background of false vacuum, where the size of a bubble is determined by balancing the energy gain proportional to the bubble volume and energy loss proportional to the bubble surface. Bubbles are typically assumed to undergo isolated quantum tunnelling events and then growing classically at a model-dependent speed^9^. The quantum process is difficult to study due to the non-perturbative nature of the dynamics. To circumvent this issue, early works have explored the possibility of directly creating new universes in a laboratory setting^10^ or in condensed-matter systems^11^. With advances in ultracold atomic gases, certain aspects of the false vacuum decay can now be studied in tabletop experiments^12^.

Recently, there has been a flurry of interest in simulating quantum field theories using synthetic platforms of ultracold atoms, superconducting circuits, trapped ions and Rydberg atoms^13–15^, with different proposals specifically addressing false vacuum decay^16–22^. Two main approaches involve either using digital quantum computers to directly emulate the quantum field theory in question or setting up an analogous system that can be initialized in the false vacuum via a controllable first-order phase transition. In this paper, we take the latter approach and set up a quantum annealer with 5,564 superconducting flux qubits, which had previously been used to study the spin glass transition^23^ and the Kibble–Zurek mechanism^24–26^. We arrange the qubits in a ring, realizing the ferromagnetic quantum Ising model. By tuning the uniform longitudinal field, we initialize the system in the metastable false vacuum state and observe the decay into the true vacuum. The discrete nature of the qubit lattice gives us a direct window into quantized bubble creation, in which a cascade of bubble sizes is seen to emerge by tuning the longitudinal field. Moreover, the longitudinal field in the quantum annealer exhibits intrinsic modulation throughout the decay, driving the dynamics and extending the regime in which we observe the same scaling laws as in coherent quantum dynamics up to 1,000 qubit time units.

Quench dynamics of the Ising chain have recently attracted much interest due to the confinement effect imposed by the longitudinal field^27–31^. The latter has direct implications for false vacuum decay, enabling analytic predictions of the decay rate^32–34^. Our simulation targets a different regime in which quantized bubbles dominate the out-of-equilibrium dynamics, originally proposed in the context of the generalized Kibble–Zurek effect^35^. This enables us to access false vacuum decay dynamics beyond the initial bubble creation and into the previously unexplored regime of interacting bubbles. We find that a large quantized bubble cannot spread in isolation, which is also the case in the semiclassical regime due to Bloch oscillations^36^. It is only through the interaction of two neighbouring bubbles that one bubble can enlarge itself by reducing the size of the other. Once reduced to the smallest size of one lattice site, the bubble can then move freely along the system. These results imply that false vacuum dynamics can be viewed as a heterogeneous gas of bubbles, where the smallest ‘light’ bubbles bounce around in the background of larger ‘heavy’ bubbles that directly interact with each other.

## Quantum simulation of false vacuum decay

We study the ferromagnetic quantum Ising model in transverse and longitudinal fields on a ring with *N* sites:

1 $$\hat{H}=-J\mathop{\sum }\limits_{j=1}^{N}{\hat{\sigma }}_{j}^{z}{\hat{\sigma }}_{j+1}^{z}-{h}_{x}\mathop{\sum }\limits_{j=1}^{N}{\hat{\sigma }}_{j}^{\,x}-{h}_{z}\mathop{\sum }\limits_{j=1}^{N}{\hat{\sigma }}_{j}^{z},$$

where ${\hat{\sigma }}^{\,\alpha }$ are the Pauli matrices; *J* > 0 is the ferromagnetic interaction between the nearest-neighbour spins; and *h*_*x*_ and *h*_*z*_ are the transverse and longitudinal fields, respectively. We apply periodic boundary conditions by identifying spin ${\hat{\sigma }}_{N+1}^{z}\equiv {\hat{\sigma }}_{1}^{z}$. The field *h*_*x*_ drives the quantum dynamics of the system, whereas *h*_*z*_ imposes an energy bias between the states |↑〉 and |↓〉.

**Fig. 1:**
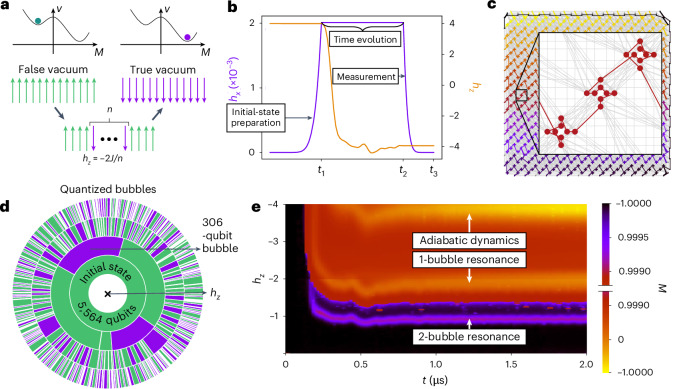
Realizing false vacuum decay on a quantum annealer. **a**, Semiclassical energy landscape *V* as a function of magnetization *M* of a ferromagnetic Ising chain in transverse (*h*_*x*_) and longitudinal (*h*_*z*_) fields. The landscape exhibits a local metastable minimum dubbed as the false vacuum, represented by the polarized |↑↑…↑〉 state. The global minimum or true vacuum is the other polarized |↓↓…↓〉 state. The false vacuum decay unfolds via the creation of quantized true vacuum bubbles of size *n*, determined by the energy balance between the surface (4*J*) and volume (2*h*_*z*_*n*) energy contributions. **b**, False vacuum decay observation protocol. We initialize all the qubits in the |↑↑…↑〉 state by setting *h*_*z*_ > 0 and adiabatically switch *h*_*x*_ from 0 to a small value (*h*_*x*_ ≪ *J*) over time *t*_1_ = 10 μs. Then, we flip the sign of *h*_*z*_, swapping the true and false vacuum states, and observing the dynamics for time *t* ≡ *t*_2_ – *t*_1_. Finally, we turn *h*_*x*_ back to 0 as fast as possible (*t*_3_ – *t*_2_ ≳ 0.18 μs) and measure the spin configuration in the ${\hat{\sigma }}^{z}$ basis. This protocol is repeated 1,000 times for each value of *t*. **c**, Embedding of a 5,564-qubit ring on the Pegasus graph of the 5,614-qubit device D-Wave Advantage_system5.4, located in Jülich, Germany. The Pegasus graph contains 15 × 15 × 3 eight-qubit Chimera cells with complete bipartite connectivity (coloured crosses) that are coupled by additional external and odd couplers (grey lines)^57^, such that each qubit is, on average, connected to 15 other qubits. Qubits within the eight-qubit cells are connected along randomly sampled one-dimensional chains (inset). **d**, Spin configurations measured in our quantum simulation. The inner ring shows the initial false vacuum state comprising 5,564 spins (for clarity, only 1,000 out of 5,564 spins in a single configuration are shown). The outer three rings show configurations measured at *h*_*z*_ = –0.1, –0.5 and –2 with *h*_*z*_ decreasing radially. An example of a large *n* = 306 quantized bubble shown in purple highlights the extent of the observed bubble sizes. **e**, Magnetization *M* heat profile versus time *t* and longitudinal-field magnitude *h*_*z*_ at transverse-field strength of *h*_*x*_ = 0.002. The colour scheme is split into two separate linear scales, a larger scale from –1 to 0.999 (bottom half) and a smaller scale from 0.999 to 1 (top half). The adiabatic dynamics and the *n* = 1-bubble resonance are easily observed on the larger scale, whereas the *n* = 2-bubble resonance can only be resolved in the fourth decimal of *M* due to the decrease in the rate of dynamics by an order of magnitude. The apparent resonance at *h*_*z*_ = –4 is identified with adiabatic dynamics rather than bubble creation, in which the system follows an instantaneous ground state during the evolution.

In the regime 0 ≤ *h*_*x*_ ≪ *J* and *h*_*z*_ = 0, there are two degenerate ground states |↑…↑〉 and |↓…↓〉. When *h*_*z*_ > 0, the |↑…↑〉 state becomes the ground or true vacuum state and |↓…↓〉, a metastable or false vacuum state (Fig. 1a). By first setting *h*_*z*_ > 0 and adiabatically turning on *h*_*x*_ ≪ *J*, we initialize the system in the |↑…↑〉 state. Then, we induce a first-order phase transition by flipping the sign of *h*_*z*_, swapping the true and false vacuas, and observe the dynamics for time *t*. Finally, we turn *h*_*x*_ back to zero as fast as possible and measure the spin configuration in the ${\hat{\sigma }}^{z}$ basis. Figure 1b illustrates the described protocol, and Fig. 1c shows the embedding of the spin chain in a qubit array used in our quantum simulations. We note here that *h*_*z*_(*t*) was experimentally determined through single-qubit measurements and exhibits large modulation around the final target value after the flip. This modulation extends up to *t* ≈ 0.75 μs in the evolution time and it plays an important role in the interpretation of our data.

Our quantum simulations are performed in the small *h*_*x*_ ≪ *J* regime, where we can apply semiclassical intuition based on the diagonal part of the Hamiltonian in the *z* basis. In this case, possible configurations of the system can be approximately organized into sectors with the same value of magnetization, $M=\langle {\sum }_{i}{\hat{\sigma }}_{i}^{z}/{N\,}\rangle$, separated by energy gaps determined by *h*_*z*_. For general values of *h*_*z*_, the initial |↑…↑〉 state stays an eigenstate in its own *M* sector after the *h*_*z*_ sign flip and no dynamics of *M* are observed. This is due to the large energy separation between different *M* sectors that cannot be hybridized by a small *h*_*x*_. However, for specific values of *h*_*z*_ = –2*J*/*n*, where *n* > 0 is an integer, the surface energy cost for flipping a domain of *n* spins, 4*J*, is exactly balanced out by the volume energy gain, 2*h*_*z*_*n* (ref. ^35^). Hence, an arbitrarily small *h*_*x*_ is sufficient to hybridize the classical computational basis states into eigenstates consisting of a superposition of the |↑…↑〉 state and so-called *n*-bubbles, that is, domain walls in the background of |↑…↑〉. For example, |↑↑↑↓↓↓↑↑↑〉 is a state with a single 3-bubble. Figure 1d shows the spin configurations measured in our quantum simulations with bubble sizes up to 306 spins, which is consistent with the theoretical prediction in which we can form increasingly larger bubbles by decreasing *h*_*z*_ according to *h*_*z*_ = –2*J*/*n*. For these discrete values of *h*_*z*_, the initial state is no longer an eigenstate and undergoes non-trivial quantum dynamics, resulting in large changes in *M*. Figure 1e shows the observed *n* = 1 and *n* = 2 resonances, where large changes in *M* can be seen at *h*_*z*_ = –2*J* and *h*_*z*_ = –*J*, respectively, in contrast to other values of *h*_*z*_.

Strong changes in *M* can also be observed (Fig. 1e) at values of *h*_*z*_ ≈ – 4*J*, where no dynamical resonances are expected. Such a large *h*_*z*_ leads to thermally assisted adiabatic dynamics^37^, where the system can follow the instantaneous ground state during time evolution. The adiabatic theorem is applicable if the timescale of Hamiltonian changes is slower than or comparable to ${t}_{\rm{a}}\propto {{{\Delta }}}_{\rm{min}}^{-2}$, where *Δ*_min_ is the minimum gap between the instantaneous ground state and the first excited state. In the case of ∣*h*_*z*_∣ ≫ *J*, *h*_*x*_, the gap becomes large enough for the timescale of *h*_*z*_(*t*) to match *t*_a_. Therefore, no bubble creation takes place and the spins turn simultaneously and in accordance with *h*_*z*_(*t*), changing the initial state from fully polarized and triggering more complex resonant processes (Supplementary Section 2).

## Observation of quantized bubbles and dynamical scaling laws

**Fig. 2:**
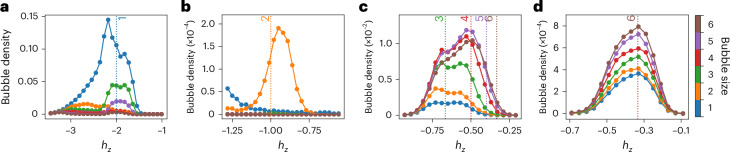
Observation of quantized bubbles. **a**–**d**, Bubble density measurements at *J* = 1 and different *h*_*z*_ magnitudes, with *h*_*x*_ = 0.002 and *t* = 2 μs (**a** and **b**), *h*_*x*_ = 0.05 and *t* = 1 μs (**c**) and *h*_*x*_ = 0.1 and *t* = 1 μs (**d**). The bubble sizes of *n* = 1, 2…6 are seen to be dominant around their respective resonances *h*_*z*_ = –2*J*/*n*, indicated by the vertical dotted lines. The times shown in **b**–**d** were chosen to allow the number of bubbles to grow and limit the impact of thermal effects, whereas the data in **a** were intentionally sampled at a time after the *h*_*z*_(*t*) modulation stops, marking the onset of thermalization. The non-monotonic 1-bubble density curve is due to faster thermalization at the resonance compared with the surrounding *h*_*z*_ values. The chosen values of *h*_*x*_ represent the cases in which we observed the most prominent resonant peaks and showcase the point that an increase of two orders of magnitude in *h*_*x*_ is required to observe higher-*n* resonances. In **b** and **c**, we probably see more than one resonance at a time due to *h*_*z*_(*t*) going through multiple *n*-bubble resonances (*h*_*z*_ = –2*J*/*n*) as it goes from a positive value to a specific resonance. This means that if we are probing the 3-bubble resonance, for example, we are also crossing the 4-, 5- and 6-bubble resonances beforehand. If we take into account additional thermalization and bubble interaction effects, there is a high likelihood of observing a few higher resonances (*n* = 4, 5 and 6), alongside *n* = 3 (for example, in **c**). The error bars across the entire figure come from counting errors on the annealer and are smaller than the size of the symbols.

To ascertain which bubbles are involved in magnetization changes, we measured the *n*-bubble density ${\lambda }_{n}=(1/N\,)\mathop{\sum }\nolimits_{i = 1}^{N}\langle {\hat{P}}_{i}^{\uparrow }[\mathop{\prod }\nolimits_{j = 1}^{n}{\hat{P}}_{i+j}^{\downarrow }]{\hat{P}}_{i+n+1}^{\uparrow }\rangle$, where ${\hat{P}}^{\sigma }=\left\vert \sigma \right\rangle \left\langle \sigma \right\vert$ is a projector on the local *σ* = {*↑*, *↓*} spin state. Figure 2a–d shows the detected 1-, 2-, 3-, 4-, 5- and 6-bubble resonances. We observe a strong suppression of all the other bubble sizes, except for the expected ones. According to the theoretical analysis presented in Methods, the leading-order effective Hamiltonian describing an *n*-bubble resonance is proportional to ${h}_{x}^{n}$. If we assume *h*_*x*_ < *J*, 1-bubbles are the fastest followed by 2-bubbles and so on, arbitrarily slowing down the dynamics as *n* increases. Figure 2a–d shows that we need to increase *h*_*x*_ by at least two orders of magnitude to begin to observe hints of higher resonances through low-density bubble formation, which is consistent with the theoretical prediction.

**Fig. 3:**
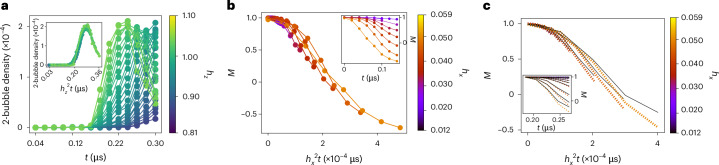
Scaling laws for bubble dynamics. **a**, 2-bubble density at *h*_*x*_ = 0.002 as a function of time at various *h*_*z*_ magnitudes (colour bar). The inset shows the collapse of different curves when time is rescaled by ${h}_{z}^{2}$ in accordance with the Landau–Zener theory^35^. **b**,**c**, Magnetization at *h*_*z*_ = –*J* resonance as a function of rescaled time ${h}_{x}^{2}t$, for different values of *h*_*x*_ indicated on the colour bar. Both the measured magnetization curves in **b** and the three-spin Bloch–Redfield numerical simulations in **c** follow the same ${h}_{x}^{2}$ scaling law, suggesting that the effective Hamiltonian governing the dynamics is proportional to ${h}_{x}^{2}$. The inset in **b** shows the raw data obtained on the quantum annealer without rescaling. The unscaled results of the numerical simulations are shown in the inset of **c**, where the black curves show the magnetization in the effective model describing the *h*_*z*_ = –*J* resonance (Methods). The error bars in **a** and **b** come from counting errors on the annealer and are smaller than the size of the symbols.

In a two-level approximation^35^, tunnelling events to different *n*-bubbles can be thought of as Landau–Zener transitions, where the metastable state |↑…↑〉 and an *n*-bubble state at the appropriate resonant conditions are the two states involved in the anticrossing. According to the Landau–Zener theory, it follows that the *n*-bubble density ${\lambda }_{n}\propto {\tau }_{{\rm{Q}}}{h}_{x}^{n}$ should be proportional to the product of the time it takes for the Hamiltonian to traverse the anticrossing *τ*_Q_, determined by *h*_*z*_(*t*) in our case, and the *n*th power of *h*_*x*_. Using our single-qubit measurements, we show that the time it takes for *h*_*z*_(*t*) to reach zero during its sign flip is proportional to the square of its magnitude (*τ*_Q_ ∝ ∣*h*_*z*_∣^2^; Supplementary Section 3). This means that *λ*_*n*_(*t*) curves measured at different pause times between the initialization and measurement ramp *t* should collapse onto a single curve if we multiply *t* by ${h}_{z}^{2}$. Figure 3a shows that the *λ*_2_ curves indeed exhibit a collapse according to this law.

Nevertheless, to fully understand the dynamics in our quantum annealer, it is necessary to account for all the dominant processes and not only the creation of bubbles. We will focus on the dynamics from the initial state |↑…↑〉, for which the creation of *n*-bubbles happens at *h*_*z*_ = –2*J*/*n*. For each resonance, we have derived the corresponding effective model using the Schrieffer–Wolff transformation^38^ and we present the effective Hamiltonians at leading orders in Methods. The effective Hamiltonian describing the dynamics at the 2-bubble resonance at *h*_*z*_ = –*J* is proportional to ${h}_{x}^{2}$. Figure 3b shows the magnetization measurements taken at this resonance using the quantum annealer and how the *M*(*t*) curves collapse when scaling the time axis with ${h}_{x}^{2}$. We have compared this result with the Bloch–Redfield numerical emulation of the quantum annealer (Fig. 3c). In contrast to the Lindblad formalism, Bloch–Redfield emulation is designed to incorporate thermalization effects and it suggests that the ${h}_{x}^{2}$ scaling law is the same as that in coherent quantum dynamics. We note that Fig. 3b shows only the initial behaviour of *M*(*t*), which follows the *h*_*z*_(*t*) modulation at later times; however, after *h*_*z*_(*t*) modulation stops, an ${h}_{x}^{3}$ scaling law emerges as a consequence of thermalization combined with a relatively slow quantum simulation measurement ramp (Supplementary Sections 6–9).

## Bubble interactions

**Fig. 4:**
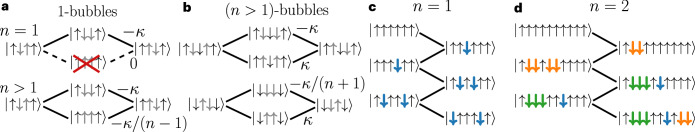
Schematic of bubble dynamics. **a**,**b**, Second-order processes for bubble hopping and interactions. The amplitude of each contributing path is shown in units of $\kappa ={h}_{x}^{2}/(2{h}_{z})=-n{h}_{x}^{2}/(4J)$. The rate of the process is given by the sum of all the paths. **a**, 1-bubbles can always hop to neighbouring sites via a second-order process. For *n* = 1, the lower path cannot be used (crossed out) as it is resonant and is, therefore, already accounted for by first-order processes. For *n* > 1, the two paths do not cancel out since one changes the number of domain walls and the other does not. **b**, In the case of larger bubbles, the two paths preserve the number of domain walls (top). Their respective amplitudes only depend on the change in the number of *↓* spins, making them opposite in sign and cancelling each other out, meaning that larger bubbles cannot directly hop. However, when next to each other, *n*-bubbles can exchange *↓* spins. The interface between them is a single *↓* spin and one of the two paths changes the number of domain walls, leading to a different amplitude (bottom). This type of spin exchange is not possible for 1-bubbles since no bubble can get smaller. For *n* > 1, these interactions lead to bubbles of size other than *n*. Through multiple consecutive exchanges, even 1-bubbles can emerge, which are then able to hop. **c**, Bubble dynamics at the *n* = 1 resonance. 1-bubbles are created, which then hop around the system; furthermore, no larger bubbles can be produced. **d**, Bubble dynamics at the *n* = 2 resonance, which is representative of all *n* > 1. 2-bubbles are created and cannot move, after which neighbouring 2-bubbles create 1- and 3-bubbles through interaction effects. Larger bubbles cannot move, whereas 1-bubbles can hop around the system. The colours of the spins in **c** and **d** correspond to the size of the bubble as in Fig. 2.

Bubble interactions play a crucial role in the dynamics at higher *n* > 1 resonances, with *h*_*z*_ = –2*J*/*n*. This can be understood intuitively from the hopping processes allowed by the energetics (Fig. 4) and more rigorously from the effective description of the dynamics presented in Methods. At the *n* = 1 resonance, 1-bubbles are initially created at rate ∝*h*_*x*_, and then hop along the chain at rate $\propto {h}_{x}^{2}/J$. However, due to the conservation of the number of bubbles (dictated by the conservation of energy), 1-bubbles cannot merge with each other to create larger bubbles. At *n* > 1 resonances, bubbles contain *n* spins and are created at rate $\propto {h}_{x}^{n}/{J}^{n-1}$. Once these *n*-bubbles are created, they cannot hop around. However, they can exchange *↓* spins with neighbouring bubbles, allowing them to change size at rate $\propto {h}_{x}^{2}/J$. This can lead to *n*-bubbles shrinking down to 1-bubbles. These then hop along the chain, restoring the flow of information. Figure 4 illustrates the stark difference between *n* = 1 and *n* > 1 in terms of allowed dynamical processes.

**Fig. 5:**
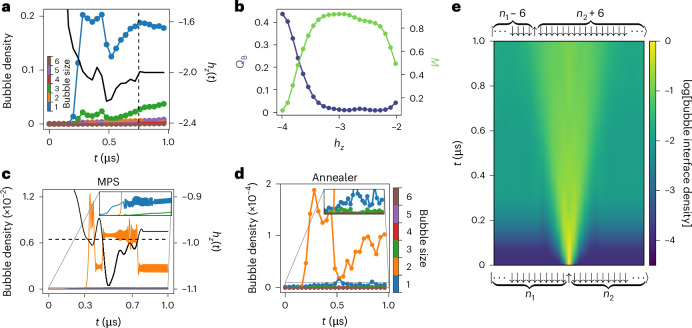
Bubble interactions. **a**, Time series measurements of the bubble density at the 1-bubble resonance (*h*_*z*_ = –2*J* and *h*_*x*_ = 0.002). During the initial *h*_*z*_(*t*) modulation, the profile of which is shown by the black curve on the right axis, the 1-bubble density (colour bar) governs the dynamics. After about ~0.75 μs (dashed line), thermalization effects take over by transforming 1-bubbles into 3- and 5-bubbles. **b**, Measurement of the emergent blockade *Q*_B_ and magnetization *M* (right axis) at *h*_*x*_ = 0.002 and *t* = 0.38 μs, plotted as a function of *h*_*z*_ magnitude. The blockade condition is violated (deviates from 0) only at *h*_*z*_ values significantly off the 1-bubble resonance (*h*_*z*_ ≤ – 3.5), accompanied by large changes in *M*. Near resonance (*h*_*z*_ ≈ – 2), even though large changes in *M* occur, the blockade condition is respected. **c**,**d**, Dynamics at the resonance *h*_*z*_ = –*J* with fixed *h*_*x*_ = 0.0203 (**c**) and *h*_*x*_ = 0.002 (**d**). MPS simulation with 100 qubits in **c** captures some of the key aspects of the data obtained on the quantum annealer in **d**. Panel **c** shows the sudden change in the number of 2-bubbles when *h*_*z*_(*t*) is exactly at the resonance point (dashed horizontal line). The inset magnifies the low-density regime, where only 1- and 3-bubbles can be seen. The increase in 1- and 3-bubbles is probably due to 2-bubbles interacting. The quantum simulation using *h*_*x*_ = 0.002 in **d** shows good agreement with the theoretical prediction in **c**. For all the measured data, the error bars come from counting errors on the annealer and are smaller than the symbol size. **e**, MPS simulation of the dynamics after an instantaneous quench from a product state shown at the bottom, containing two large bubbles (*n*_1_ = 23 and *n*_2_ = 24 spins) next to each other in a system with a total of 50 spins. The system undergoes coherent evolution with fixed *h*_*z*_ = –1 and *h*_*x*_ = 0.02, and the colour bar shows the ‘bubble interface density’, $\langle {\hat{P}}_{j-1}^{\downarrow }{\hat{P}}_{j}^{\uparrow }{\hat{P}}_{j+1}^{\downarrow }\rangle$, on a log scale and for all sites *j*. The moving front corresponds to the two bubbles exchanging *↓* spins and changing their sizes. The final state at the end of the evolution is a quantum superposition, with one of the classical configurations shown at the top.

The measured dynamics of different bubble sizes at *h*_*z*_ = – 2*J* resonance (Fig. 5a) is indeed consistent with the picture that 1-bubbles remain approximately quantized and do not grow with time. On the quantum annealer, this persists until thermalization kicks in and 1-bubbles start to transform into 3- and 5-bubbles, with 2- and 4-bubbles remaining suppressed throughout the time evolution. The exploration of this peculiar thermalization effect is beyond the scope of this work, as thermalization and bubble interaction effects cannot be easily separated from each other in our quantum annealer due to decoherence effects.

This impossibility for 1-bubbles to grow at the *n* = 1 resonance dictates that there can never be two *↓* spins next to each other in this regime. The system, therefore, experiences an emergent kinetic constraint, reminiscent of the Rydberg blockade phenomenon^39^. We quantify the blockade by measuring the operator ${\hat{Q}}_{\rm{B}}=(1/N){\sum }_{j}{\hat{P}}_{j}^{\downarrow }{\hat{P}}_{j+1}^{\downarrow }$, which counts the density of neighbouring *↓* spins. We expect $\langle {\hat{Q}}_{\rm{B}}\rangle$ to be strongly suppressed at around *h*_*z*_ = –2*J*, rising towards 0.5 in other dynamical settings. Figure 5b shows a good match between these predictions and quantum simulation data. Meanwhile, the total magnetization strongly deviates from the initial value of 1, showing that the lack of neighbouring excitations is not trivially due to frozen dynamics.

Our theoretical predictions imply that in a quantum simulation tuned to an *n* > 1 resonance, the size of the bubbles is not limited to *n*, even if the system is perfectly isolated from the environment. This can be seen in a fully coherent matrix-product state (MPS) simulation of a system with *N* = 100 spins at *n* = 2 resonance (Fig. 5c). Although 2-bubbles dominate, 1- and 3-bubbles are also visible. This is expected as they are produced by the interactions of 2-bubbles. A qualitatively similar behaviour is also seen in the quantum simulation data (Fig. 5d).

The data in Fig. 5c,d also highlights another important property for *n* > 1: the number of 2-bubbles changes abruptly at some times and stays approximately constant during the rest of the simulation. The timings of abrupt changes coincide exactly with *h*_*z*_(*t*), hitting the appropriate resonant value, whereas the rest of the time, the system is slightly away from resonance. This highlights the sensitivity to detuning, *δ* = *h*_*z*_ + 2*J*/*n*, which competes with ${h}_{x}^{n}/{J}^{n-1}$. As *h*_*x*_/*J* ≪ 1, even a small *δ* is enough to overpower the bubble creation terms for *n* > 1. As the detuning is a diagonal contribution, it leads to the suppression of all dynamical processes, including bubble creation. This pattern of sudden changes due to fluctuation in *h*_*z*_ is clearly captured in the numerical simulation shown in Fig. 5c, but it is also visible in the annealer data shown in Fig. 5d. We note that this sensitivity to detuning is expected to be less strong for *n* = 1, as in that case, *δ* only competes with *h*_*x*_.

To further highlight the importance of bubble interactions, we have studied a closed system with two large bubbles next to each other, essentially occupying the entire system (Fig. 5e). This setup allows us to study a single boundary as the other boundaries are too far to play a role during the simulation time and there is no room for new bubbles to appear; hence, the only active process is the exchange between the two bubbles. We can then track the interface between them by measuring ${\hat{P}}_{j-1}^{\downarrow }{\hat{P}}_{j}^{\uparrow }{\hat{P}}_{j+1}^{\downarrow }$, which is plotted on a log scale in Fig. 5e at the *h*_*z*_ = –*J* resonance. Although the interface density is 1 at a single location at time *t* = 0 and zero everywhere else, as time goes on, the interface steadily delocalizes due to the bubbles exchanging *↓* spins and consequently changing their sizes. We expect similar behaviour to hold at other *n* > 1 resonances.

## Discussion and outlook

We have performed a quantum simulation of the false vacuum decay and identified its underlying mechanism—the formation of quantized bubbles of true vacuum. These results are consistent with the standard scenario in which the size of the formed bubble is determined by the competition between the volume energy gain and surface energy loss. Our central finding is that interactions between bubbles are the key next-order effect after bubble creation. The understanding of bubble interactions is, therefore, crucial for a comprehensive description of false vacuum decay, not only in microscopic models such as the one studied here but also in quantum field theory and cosmological models of the Big Bang.

Previous studies^32,33^ have explored a different parameter regime in which *h*_*x*_ is not small and the energy spectrum forms a continuum. Although the possibility of resonances was pointed out as a subleading effect^32^, these analytical considerations still assume a dilute bubble picture, neglecting interactions between bubbles. The experimental study^12^ corroborated these predictions in a bosonic gas of ^23^Na atoms. Although this system is believed to exhibit the same critical behaviour as the Ising model, its microscopic continuum nature is markedly different from our lattice realization. Moreover, the experiment^12^ probed a different regime of large *h*_*x*_ ≲ *J*, where the main observable signature is the exponential decay rate of the metastable false vacuum, in contrast to our small *h*_*x*_ ≪ *J* regime that allows for the in situ monitoring of bubble formation and growth.

Our work showcases the usefulness of current quantum annealing devices in probing complex many-body dynamics. This is demonstrated here through the observation of large bubbles of up to 300 spins embedded in a 5,564-qubit system, with their dynamics monitored over 1,000 individual qubit time units, even without the fast annealing capability^25,26^. The essential aspects of small bubble formation and interactions in one dimension were successfully captured by our tensor network simulations and effective models, providing a proof of principle that quantum annealers can be used to study such complex many-body phenomena. With recent advances in fast annealing, quantum annealers have been argued to outperform classical simulations on certain problems^40^; thus, they could provide a powerful tool for the exploration of false vacuum decay in higher dimensions and various lattice topologies, potentially reaching classically intractable computational complexity.

Last, let us mention a few examples of other non-equilibrium phenomena that can be accessed in the platform established here. False vacuum decay, as a specific instance of a first-order quantum phase transition, allows to probe generalizations of the Kibble–Zurek scaling laws^11,41–43^ in such transitions. Quantum metastability—the cornerstone of the false vacuum decay phenomenon—also underlies the reaction rate theory^44–49^, allowing the use of quantum simulation for estimating the transition rate of decay processes from a metastable minimum to a lower-energy state in the presence of temperature, which is challenging to compute otherwise. In the regime of stronger longitudinal fields, confinement effects are expected to become important, possibly localizing bubbles in space and giving rise to an emergent prethermalization regime^50^. Finally, at the 1-bubble resonance, our model displays an emergent kinetic constraint that maps exactly to the so-called PXP model^51,52^ (Methods), which hosts quantum many-body scars^39,53,54^, and possibly other types of ergodicity breaking, such as Hilbert-space fragmentation and many-body localization^55,56^. This opens the way to probe non-ergodic phenomena in large systems in the presence of dissipation and potentially new types of scar in constrained models at other *n* > 1 resonances.

## Methods

### Quantum simulation on D-Wave’s quantum annealer

Our quantum simulations utilized D-Wave’s quantum annealing device Advantage_system5.4, located in Jülich, Germany, which features *N*_q_ = 5,614 qubits and is kept at a cryostat temperature of 16.4 ± 0.1 mK. The annealer implements the Hamiltonian

2 $${\hat{H}}_{{\rm{DW}}}=-\frac{A(s)}{2}\left(\mathop{\sum }\limits_{i=1}^{{N}_{q}}{\hat{\sigma }}_{i}^{\,x}\right)+\frac{B(s)}{2}\left(g(t)\mathop{\sum }\limits_{i=1}^{{N}_{q}}{h}_{i}{\hat{\sigma }}_{i}^{z}+\mathop{\sum }\limits_{i < j}^{{N}_{q}}{J}_{ij}{\hat{\sigma }}_{i}^{z}{\hat{\sigma }}_{j}^{z}\right),$$

where ${\hat{\sigma }}_{i}^{\,x,z}$ are the Pauli matrices for the *i*th qubit, *h*_*i*_ is the longitudinal external field at qubit *i* and *J*_*ij*_ are the couplings between qubits *i* and *j*, which are non-zero and user-tunable only if they are physically connected in the quantum processing unit (Fig. 1c). *A*(*s*) and *B*(*s*) represent the energy scales of their respective terms and are driven in time by the annealing schedule *s*(*t*), which is linearly interpolated from a series of user-specified points [(*t*_*i*_, *s*_*i*_)]. Similarly, *g*(*t*) is used in combination with *h*_*i*_ to manipulate the external longitudinal field in time by specifying a series of points [(*t*_*i*_, *g*_*i*_)].

Finding a ring embedding in a given graph is an instance of an NP-complete Hamiltonian circuit problem^58^. We generate our ring embedding on 5,564 qubits of the Advantage_system5.4 graph by first connecting all eight-qubit Chimera cells in the Pegasus topology (Fig. 1c). We start in the top-left corner and proceed horizontally, changing the horizontal direction at the end of every row, until we reach the bottom-right corner. The chain of qubits within each eight-qubit Chimera cell is chosen along a random suitable path (Fig. 1c, inset). The ring is closed by proceeding along the outer qubits at the right and top edge of the graph (Fig. 1c, black part of the chain). This procedure yields a ring of 5,446 qubits. We then iteratively add qubits to the chain from the set of omitted remaining qubits by adding detours into the ring until we obtain the final 5,564-qubit closed chain. We note that a few of the qubits and couplers in the full Pegasus graph are not present on the device due to fabrication defects; these are accounted for individually.

We are interested in probing the dynamics of $\hat{H}$ in equation (1) at a certain value of *h*_*x*_. We choose uniform *h*_*i*_ = *h*, *J*_*ij*_ = –1 and instantaneous values of the fields are determined from *h*_*x*_ = *A*(*s*)/*B*(*s*) and *h*_*z*_ = –*g*(*t*)*h*. At the beginning of the annealing schedule (*s*(0) = 1), we specify the initial state for all the qubits as the product state |↑…↑〉. Then, within the initial ramp time *t*_1_, we bring the system to the desired *h*_*x*_ value, which drives the dynamics we are interested in, and keep it constant for time *t* ≡ *t*_2_ – *t*_1_. Finally, we bring *h*_*x*_ to 0 within time *t*_3_ – *t*_2_, which constitutes a measurement. Only after *h*_*x*_ is brought back to 0, it is possible to read out the state of the qubits in the computational or ${\hat{\sigma }}^{z}$ basis. In summary, our annealing schedule is specified according to $[(0,1),({t}_{1},{s}_{{h}_{x}}),({t}_{2},{s}_{{h}_{x}}),({t}_{3},1)]$, where ${s}_{{h}_{x}}$ is obtained from the relation ${h}_{x}=A({s}_{{h}_{x}})/B({s}_{{h}_{x}})$. Typical timescales that we used on the D-Wave device are *t*_1_ = 10 μs, *t*_3_ – *t*_2_ = 272 ns and *t* ranging from 0 to 2 μs. After the initial-state preparation, the system remains in the |↑…↑〉 state due to the small values of *h*_*x*_ compared with *h*_*z*_. During the entire time evolution, which lasts for time *t*_3_, the system is subject to open-system dynamics, governed by two main effects: measurement by the environment and thermalization. Our single-spin measurements show that measurement by the environment is dominant whenever the system is being driven by the longitudinal external field *h*_*z*_. Whenever *h*_*z*_ becomes constant, thermalization effects become more evident and are heavily dependent on the value of *h*_*x*_, which drives the quantum dynamics of the system (Supplementary Sections 7 and 9).

### Simulations of thermalization dynamics

To capture the thermalization effects on the system’s dynamics, we employed the Bloch–Redfield master equation^59^

3 $$\frac{\rm{d}}{{\rm{d}}t}{\rho }_{ab}(t)=-{\rm{i}}{\omega }_{ab}{\rho }_{ab}(t)+\mathop{\sum }\limits_{cd}^{\rm{sec}}{R}_{abcd}{\rho }_{cd},$$

where $\hat{\rho }$ is the density matrix of the system and *ω*_*ab*_ ≡ *ω*_*a*_ – *ω*_*b*_, with *ω*_*a*_ = *E*_*a*_/*ℏ* and *E*_*a*_ being the eigenenergies of the system. sec denotes the secular approximation, which states that we can neglect all fast-rotating terms in the sum, and *R*_*abcd*_ is the Bloch–Redfield tensor^59^

4 $$\begin{array}{l}{R}_{abcd}=-\frac{1}{2{\hslash }^{2}}\sum _{\alpha ,\beta }\left\{{\delta }_{bd}\sum _{n}{A}_{an}^{\alpha }{A}_{nc}^{\beta }{S}_{\alpha \beta }({\omega }_{cn})-{A}_{ac}^{\alpha }{A}_{db}^{\beta }{S}_{\alpha \beta }({\omega }_{ca})\right.\\\left.\qquad\qquad+\,{\delta }_{ac}\sum _{n}{A}_{dn}^{\alpha }{A}_{nb}^{\beta }{S}_{\alpha \beta }({\omega }_{dn})-{A}_{ac}^{\alpha }{A}_{db}^{\beta }{S}_{\alpha \beta }({\omega }_{db})\right\},\end{array}$$

where ${A}_{ab}^{\alpha }$ are the matrix elements in the system’s eigenbasis of the operator *A*^*α*^ that couples bilinearly to the bath. Here we choose ${A}^{\alpha }={\sigma }_{\alpha }^{z}$, where *α* runs through all the spins of the system. *S*_*αβ*_(*ω*) = *ηωθ*(*ω*)exp(*ω*/*ω*_c_) is the noise power spectrum of the bath, chosen to be ohmic in our case, where *θ*(*ω*) is the Heaviside step function, *η* is the coupling strength of the system-bath coupling that ranges from 0.1 to 0.2 in our case and *ω*_c_ is the cutoff frequency higher than any other relevant energy scale.

The numerical simulations shown in Fig. 5c,e were performed under the assumption of a closed system using the MPS formalism^60^. For efficiency, the simulated system has open-boundary conditions, but we discard the boundary sites when computing observable expectation values to minimize the boundary effects. To reach the long times required for the simulation, a fourth-order time-evolving block decimation was used^61,62^. For Fig. 5c, the time step is δ*t* = 0.01 and the maximum MPS bond dimension is *χ* = 128, which was never saturated during the simulation. For Fig. 5e, the time step is *t* = 0.05 and the maximum bond dimension is *χ* = 200.

### Effective models at different resonances

To fully understand the dynamics beyond bubble creation in the vicinity of resonances, we have derived the corresponding effective Hamiltonians using the Schrieffer–Wolff transformation^38^. We quote the main results here, and the derivation and detailed analysis of the models are provided in Supplementary Section 5. For *n* = 1, in the sector containing the state |↑…↑〉, the combined effective Hamiltonian at the first and second order reads

5 $$\begin{array}{l}{\hat{H}}_{{\rm{eff}},n = 1}^{(1,2)}=-{h}_{x}\mathop{\sum }\limits_{j=1}^{N}{\hat{P}}_{j-1}^{\uparrow }{\hat{\sigma }}_{j}^{\,x}{\hat{P}}_{j+1}^{\uparrow }-\delta \mathop{\sum }\limits_{j=1}^{N}{\hat{\sigma }}_{j}^{z}\\\qquad\qquad+\,\frac{{h}_{x}^{2}}{4J}\left[\mathop{\sum }\limits_{j=1}^{N}{\hat{P}}_{j-1}^{\uparrow }\left({\hat{\sigma }}_{j}^{+}{\hat{\sigma }}_{j+1}^{-}+{\hat{\sigma }}_{j}^{-}{\hat{\sigma }}_{j+1}^{+}\right){\hat{P}}_{j+2}^{\uparrow }\right.\left.+\,2\mathop{\sum }\limits_{j=1}^{N}{\hat{P}}_{j}^{\downarrow }-\frac{3}{2}\mathop{\sum }\limits_{j=1}^{N}{\hat{P}}_{j-1}^{\downarrow }{\hat{P}}_{j+1}^{\downarrow }\right],\end{array}$$

where *δ* = *h*_*z*_ + 2*J* is the (weak) detuning away from the *n* = 1 resonance, ${\hat{\sigma }}^{\pm }=({\hat{\sigma }}^{x}\pm {\rm{i}}{\hat{\sigma }}^{\,y})/2$ are the standard spin-raising and spin-lowering operators, and ${\hat{P}}^{\downarrow }=\left\vert \downarrow \right\rangle \left\langle \downarrow \right\vert ,{\hat{P}}^{\uparrow }=\left\vert \uparrow \right\rangle \left\langle \uparrow \right\vert$ are local spin projectors.

The dynamics generated by the Hamiltonian in equation (5) can be understood as follows. The ${\hat{P}}^{\uparrow }{\hat{\sigma }}^{x}{\hat{P}}^{\uparrow }$ term allows the creation of single-site bubbles (that is, single *↓* spins in a background of *↑* spins), whereas the ${\hat{P}}^{\uparrow }\left({\hat{\sigma }}^{+}{\hat{\sigma }}^{-}+{\hat{\sigma }}^{-}{\hat{\sigma }}^{+}\right){\hat{P}}^{\uparrow }$ allows these bubbles to hop around. A sequence of allowed processes is illustrated in Fig. 4c. Importantly, due to the projectors, the bubbles cannot merge to form larger ones. This is also impossible to do using higher-order processes. A simple argument is that there are no states with larger bubbles at the same classical energy (that is, the energy contribution of the ${\hat{\sigma }}^{z}$ terms) as the |↑↑↑⋯〉 state; therefore, it is impossible to reach these states resonantly.

In the main text, we have demonstrated that one measurable consequence of the effective Hamiltonian in equation (5) is a robust emergent kinetic constraint reminiscent of the Rydberg blockade^39^. The quality of this emergent blockade can be assessed using the operator ${\hat{Q}}_{\rm{B}}$ introduced in the main text, which measures the density of neighbouring *↓* spins and can be equivalently expressed in the spin language as ${\hat{Q}}_{\rm{B}}=1/4+(1/(4N\,)){\sum }_{j}{\hat{\sigma }}_{j}^{z}{\hat{\sigma }}_{j+1}^{z}-(1/(2N\,)){\sum }_{j}{\hat{\sigma }}_{j}^{z}$.

For *n* > 1 resonances, the bubble creation term is no longer dominant as it happens at order *n* according to

6 $${\hat{H}}_{{\rm{eff}},n}={c}_{n}\frac{{h}_{x}^{n}}{{J}^{n-1}}\mathop{\sum }\limits_{j=1}^{N}{\hat{P}}_{j}^{\uparrow }\left(\mathop{\prod }\limits_{k=1}^{n}{\hat{\sigma }}_{j+k}^{-}\right){\hat{P}}_{j+n+1}^{\uparrow }+{\rm{h.c.}},$$

where *c*_*n*_ is a coefficient that depends on the multiple subprocesses involved, for example, we have *c*_2_ = –1 and *c*_3_ = –81/64. Instead, regardless of *n*, there are always other terms at order one and two that read

7 $$\begin{array}{ll}{\hat{H}}_{{\rm{eff}},n = 2}^{(1,2)}=\,-\delta \mathop{\sum }\limits_{j=1}^{N}{\hat{\sigma }}_{j}^{z}+\frac{{h}_{x}^{2}n}{4J}\mathop{\sum }\limits_{j=1}^{N}\left(\frac{{\hat{P}}_{j-1}^{\downarrow }{\hat{\sigma }}_{j}^{z}{\hat{P}}_{j+1}^{\downarrow }}{n+1}+{\hat{P}}_{j-1}^{\uparrow }{\hat{\sigma }}_{j}^{z}{\hat{P}}_{j+1}^{\downarrow }+{\hat{P}}_{j-1}^{\downarrow }{\hat{\sigma }}_{j}^{z}{\hat{P}}_{j+1}^{\uparrow }-\frac{{\hat{P}}_{j-1}^{\uparrow }{\hat{\sigma }}_{j}^{z}{\hat{P}}_{j+1}^{\uparrow }}{n-1}\right)\\\qquad+\,\frac{{h}_{x}^{2}{n}^{2}}{4J(n-1)}\mathop{\sum }\limits_{j=1}^{N}{\hat{P}}_{j-1}^{\uparrow }\left({\hat{\sigma }}_{j}^{+}{\hat{\sigma }}_{j+1}^{-}+{\hat{\sigma }}_{j}^{-}{\hat{\sigma }}_{j+1}^{+}\right){\hat{P}}_{j+2}^{\uparrow }-\,\frac{{h}_{x}^{2}{n}^{2}}{4J(n+1)}\mathop{\sum }\limits_{j=1}^{N}{\hat{P}}_{j-1}^{\downarrow }\left({\hat{\sigma }}_{j}^{+}{\hat{\sigma }}_{j+1}^{-}+{\hat{\sigma }}_{j}^{-}{\hat{\sigma }}_{j+1}^{+}\right){\hat{P}}_{j+2}^{\downarrow }.\end{array}$$

The terms on the second line create dynamics: the first term leads to 1-bubbles hopping (similar to *n* = 1), whereas the second one allows larger bubbles to exchange *↓* spins and consequently grow or shrink. The latter process allows, for example, two bubbles of order *n* to modify their size via (*n*, *n*)→(*n* – 1, *n* + 1), even if the two bubbles cannot move on their own. In the extreme case, this includes the possibility of a large bubble shrinking all the way down to a 1-bubble, which is then free to move on its own. A sequence of these allowed processes is illustrated in Fig. 4d for *n* = 2, with higher *n* values displaying qualitatively similar behaviours. As a result of the processes in equation (7), bubbles of different sizes (≤*n*) coexist in the regime we probe; in particular, we do not observe an ‘avalanche’ effect that would result in a preponderance of 1-bubbles.

Two comments are in order. First, the dynamics at *n* > 1 resonances are clearly much richer than at *n* = 1. Indeed, although the bubble interaction term should also be present for *n* = 1, it cannot act between two 1-bubbles. This would require one of them to shrink to 0, which is not resonant. Thus, in the sector of the |↑…↑〉 state in which only 1-bubbles appear, the bubble interaction term vanishes. Second, it is worth noting that the first term of the effective Hamiltonian at the *n* = 1 resonance (equation (5)), up to a global spin flip, is identical to the PXP model used to describe chains of Rydberg atoms^51,52^. The second term can then be recast as $-2\delta {\sum }_{j}{\hat{P}}_{j}^{\downarrow }$ up to an irrelevant constant, and then becomes the chemical potential for the effective PXP model. On the other hand, to the best of our knowledge, the effective Hamiltonians for *n* > 1 resonances (equation (7)) do not map to the models previously studied in the literature.

## Online content

Any methods, additional references, Nature Portfolio reporting summaries, source data, extended data, supplementary information, acknowledgements, peer review information; details of author contributions and competing interests; and statements of data and code availability are available at 10.1038/s41567-024-02765-w.

## Supplementary information

Supplementary Information Supplementary Figs. 1–20, Sections 1–9, discussion and derivations.

## Data Availability

The data supporting the plots within this paper and other findings of this study are available at 10.5518/1579.

## Code availability

The code used to obtain the data from the quantum annealer is available at 10.5518/1579.
