# Supplementary material for: Stirring the false vacuum via interacting quantized bubbles on a 5,564-qubit quantum annealer
Source: Nat Phys. 2025 Feb 4;21(3):386–92. doi: 10.1038/s41567-024-02765-w (PMC11908970; doi:10.1038/s41567-024-02765-w)
Supplement: Supplementary file 1 — Supplementary Figs. 1–20, Sections 1–9, discussion and derivations. [file 41567_2024_2765_MOESM1_ESM.pdf]

nature physics

Supplementary information

https://doi.org/10.1038/s41567-024-02765-w

Stirring the false vacuum via interacting quantized bubbles on a 5,564-qubit quantum annealer

In the format provided by the authors and unedited

Supplementary Information

CONTENTS

I. The model 1

II. More complex resonant processes 1

III. Single-spin measurements and the Lindblad master equation 2
A. Measurement protocol 2
B. Modeling magnetization dynamics via Lindblad master equation 3

IV. Numerical emulation of magnetization dynamics on the D-Wave quantum annealer 4
A. Bloch-Redfield emulation 4
B. Coherent emulation 5

V. Effective models at different resonances 7
A. $h_z = -2J$ resonance: the PXP model 7
B. $h_z = -J$ resonance 9
C. $n > 2$ resonances 10

VI. Quantum simulation of the $h_z = -2J$ resonance 11

VII. Numerical emulation of the $h_z = -2J$ resonance 12

VIII. Quantum simulation of the $h_z = -J$ resonance 13

IX. Numerical emulation of the $h_z = -J$ resonance 14

Supplementary References 15

I. THE MODEL

To contextualize the following sections and set the notation, here we briefly summarize the model from the main text and our observation protocol on the quantum annealer. We study the ferromagnetic Ising model on a ring with N spins, described by the Hamiltonian

$$\hat{H} = -\sum_{j=1}^{N} J\hat{\sigma}_j^z\hat{\sigma}_{j+1}^z - h_x\sum_{j=1}^{N}\hat{\sigma}_j^x - h_z\sum_{j=1}^{N}\hat{\sigma}_j^z, \quad (1)$$

where $\hat{\sigma}^\alpha$ are the standard Pauli matrices, $J > 0$ is the ferromagnetic interaction strength between nearest-neighbor spins (unless specified otherwise, we will assume $J = 1$), h_x and h_z are the transverse and longitudinal fields, respectively, and periodic boundary conditions (PBCs) are applied by identifying spin $N + 1 \equiv 1$. The field h_x is responsible for driving the quantum dynamics of the system, while h_z imposes an energy bias between the states $|\uparrow\rangle$ and $|\downarrow\rangle$.

In the regime $0 < h_x \ll J$ and $h_z = 0$, there are two degenerate ground states which are approximately given by product states $|\uparrow ... \uparrow\rangle$ and $|\downarrow ... \downarrow\rangle$. When $h_z > 0$, the $|\uparrow ... \uparrow\rangle$ state becomes the ground or true vacuum state, while $|\downarrow ... \downarrow\rangle$ is raised in energy and becomes a metastable or false vacuum state. By first setting $h_z > 0$ and adiabatically turning on h_x to a small (positive) value, $h_x \ll J$, we initialize the system in the $|\uparrow ... \uparrow\rangle$ product state. Then we flip the sign of h_z, switching the true and false vacuum states and observing the dynamics for a time duration t. Finally, we turn h_x back to 0 as fast as possible and measure the spin configuration in the computational or $\hat{\sigma}^z$ basis. A typical observable that will be extracted from such measurements is the spin magnetization along the z-axis,

$$M(t) = \frac{1}{N}\sum_i \langle\psi(t)|\sigma_i^z|\psi(t)\rangle. \quad (2)$$

We note that single-qubit measurements on the actual device show that h_z typically displays more complex behavior than the idealized case described above. For example, we find that $h_z(t)$ can exhibit large modulation around the final target value after the flip. If the magnitude of h_z is large, this can trigger different behavior from the one we focused on in the main text. These more complex resonant processes, resulting from the ground state being different from $|\uparrow \ldots \uparrow\rangle$, are discussed in Sec. II below, while the characterization of the h_z field modulation is the subject of Sec. III.

II. MORE COMPLEX RESONANT PROCESSES

Supplementary Figure 1 shows the normalized n-bubble density measurements at time $t = 2\mu s$, between the initialization and measurement ramps, as we vary h_z at a fixed $h_x = 0.002$. The n-bubble density is characterized by the following operator introduced in the main text:

$$\lambda_n = \frac{1}{N}\sum_{i=1}^{N}\langle\hat{P}_i^{\uparrow}[\prod_{j=1}^{n}\hat{P}_{i+j}^{\downarrow}]\hat{P}_{i+n+1}^{\uparrow}\rangle\,, \quad (3)$$

where $\hat{P}^\sigma = |\sigma\rangle\langle\sigma|$ is a projector on the $\sigma = \uparrow,\downarrow$ spin state.

We observe a clear suppression of 1-bubbles when h_z drops below the 1-bubble resonance at $h_z = -2$. For example, we observe additional peaks at $h_z \approx -2.65, -3.2$ that were briefly mentioned in the main text. We interpret these peaks as more complex resonant processes that occur in a different regime, where the dynamics are dominated by large $|h_z|$ (i.e., not associated with bubble formation discussed in the main text). Specifically, in

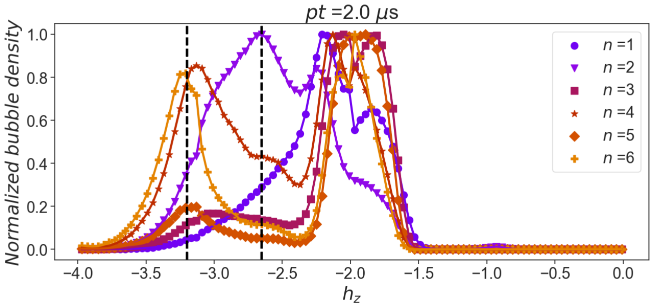

Supplementary Figure 1. Measurements of normalized n-bubble density, Eq. (3), up to $n = 6$ at time $pt = 2\mu s$, between the initialization and measurement ramps. We fix $h_x = 0.002$ and vary h_z magnitude. The bubble density is normalized with respect to the largest density we measured: $\lambda_1^{max} = 0.144$, $\lambda_2^{max} = 0.016$, $\lambda_3^{max} = 0.045$, $\lambda_4^{max} = 0.006$, $\lambda_5^{max} = 0.02$, and $\lambda_6^{max} = 0.004$. There is a clear peak of 2- and 4-bubble density at $h_z \approx -2.65$, followed by a peak of 4- and 6-bubble densities at $h_z \approx -3.2$, both marked by black dashed lines. They are most likely due to more complex resonant processes (see text for details).

Supplementary Figure 1 we see 2- and 4-bubble densities peak at $h_z \approx -2.65$, as well as 4- and 6-bubble densities peak at $h_z \approx -3.2$. This is in contrast to the 1-bubble resonance, where we observe only odd bubble densities (1,3,5). We conjecture that the $h_z \approx -2.65, -3.2$ peaks stem from more complex resonant processes occurring due to the initial state not being exactly the uniformly polarized false vacuum state. At these high magnitudes of $|h_z|$, the system can be forced adiabatically into a sector of states whose classical energy is different from that of the $|\uparrow \ldots \uparrow\rangle$ state due to stronger driving of the $h_z(t)$ field. This is in contrast to the 1- and 2-bubble resonances at $h_z = -2, -1$ where the initial state remains the $|\uparrow \ldots \uparrow\rangle$ state throughout the initial $h_z(t)$ drive. The n-bubble resonances picture presented in the main text is based on the initial state being the $|\uparrow ... \uparrow\rangle$ state. If this is not the case, many more resonant processes become possible, some of which can be seen in Supplementary Figure 1.

III. SINGLE-SPIN MEASUREMENTS AND THE LINDBLAD MASTER EQUATION

We have performed single-spin measurements on the D-Wave *Advantage_system*5.4 to gain insight into the behavior of the actual $h_z(t)$ that is applied to the qubits, as well as to quantify the degree of coherence of the device and precision of the quantum simulation protocol in the main text.

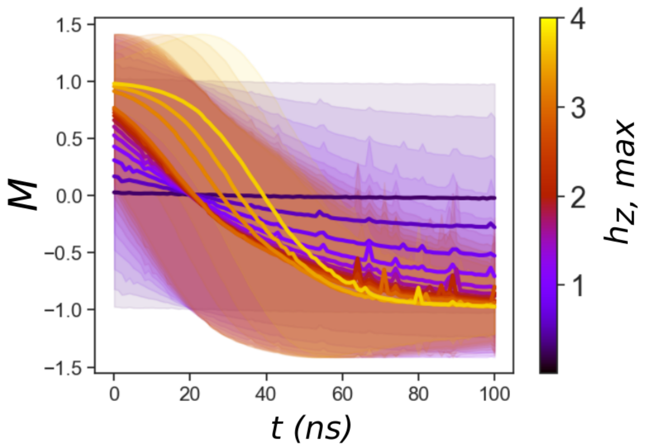

Supplementary Figure 2. Single-spin measurements of $M(t)$ on the quantum annealer with $J = 0$ and $h_{x,\max} = 0.1$, averaged across all 5564 spins, each sampled 10^3 times to be directly compared with its Lindblad emulation shown in Supplementary Figure 4 below. The $h_{z,\max}$ values corresponding to different curves are shown in the color bar on the right. When $h_{z,\max}$ is less than 1, the M curves saturate to values between -1 and 0. The shaded areas around each curve represent the standard deviation obtained from averaging the data and have the same color as the corresponding curve.

A. Measurement protocol

Our measurement protocol involves first setting to zero all the couplings between qubits on the device, $J = 0$. We then initialize all available 5564 qubits on the device in the $|\uparrow\rangle$ state. The field h_z is tunable in the range from 0 to 4, and we initially set it to $h_z(0) = h_{z,\max}$. Then, we adiabatically bring h_x to some value that we will call $h_{x,\max}$ (below we will use $h_{x,\max} = 0.1$ as an example) in time *irt*. At that point, we switch $h_z(t)$ as fast as the device allows, which is still adiabatic, to $h_z(irt + pt + mt) = h_{z,\min}$ (typically, we take $h_{z,\min} = -h_{z,\max}$). We pause h_x for a time *pt* and then bring it back to 0 in time *mt*, which constitutes a measurement. At the end of the protocol, we read out the states of all the qubits in the computational or $\hat{\sigma}^z$ basis. We perform 10^3 simultaneous measurements on 5564 qubits and average over both to get the average z-magnetization M and its standard deviation.

In all the plots below, unless specified otherwise, *pt* is replaced with t for simplicity. Moreover, in this and the following section, we will generally be careful to distinguish the instantaneous values of the fields, h_x and h_z, from their magnitudes, $h_{x,\max}$ and $h_{z,\max}$, as our goal is to understand in detail the intrinsic behavior of the annealer that may differ from our idealized model (1). To minimize clutter, in other sections will frequently drop the subscript "max" when referring to the field magnitudes if there is no risk of confusion.

Supplementary Figure 2 shows the measured $M(t)$ [Eq. (2)] and its standard deviation. As $h_{z,\max}$ increases

from 0 to 4, the $M(t)$ curves change from being completely flat around 0 (small $h_{z,\max}$) to a sigmoid-like function between 1 and -1 (large $h_{z,\max}$). These results cannot be explained only in terms of coherent adiabatic dynamics, even if we couple the system to an external bath that causes the spins to thermalize into the ground state. In both cases, we would expect every spin to follow the instantaneous ground state, which means that at $t = 100ns$ the spins should predominately be in the $|\downarrow\rangle$ state, where $M = -1$, which is not the case. When the magnitude of $h_z(t)$ is less than 1, M saturates to some h_z-dependent value between -1 and 0.

B. Modeling magnetization dynamics via Lindblad master equation

Our coherent (Supplementary Figure 7) and Bloch-Redfield (not shown) emulations of single spin dynamics show that different saturation points of M cannot be reproduced in a closed system, unless the measurement ramp is significantly reduced in a physically implausible manner, suggesting that the environment is effectively performing measurements on the system and collapsing the wave function onto the σ^z basis before we measure the system on the quantum annealer. Otherwise, the system simply follows the instantaneous ground state throughout the entire time evolution. Therefore, to model single spin dynamics measured on the device, we require open system dynamics in the form of a Lindblad master equation

$$\dot{\hat{\rho}} = -\frac{i}{\hbar}[\hat{H}(t), \hat{\rho}(t)] + \\ + \sum_n \frac{1}{2}\left[2\hat{C}_n\hat{\rho}(t)\hat{C}_n^\dagger - \hat{\rho}(t)\hat{C}_n^\dagger\hat{C}_n - \hat{C}_n^\dagger\hat{C}_n\rho(t)\right], \quad (4)$$

where $\hat{C}_n = \sqrt{\gamma}\hat{\sigma}_n^z$ are the collapse operators, which couple the $\hat{\sigma}^z$ operator of the system to the environment and $\gamma_n = \gamma$ is the corresponding rate. Using this choice of environmental coupling we can interpret the impact of the environment as random measurements of each of the spins in the computational basis at a rate γ.

Supplementary Figure 3 showcases the effect of the environment on the system: the magnetization of a single spin now clearly deviates from the ground-state expectation value as γ is increased, which is not the case during either coherent or thermally assisted adiabatic evolution.

Supplementary Figure 4 shows the best match of the Lindblad emulation to the M dynamics measured in the quantum simulation in Supplementary Figure 2. We used $\gamma = 0.0002\mu s^{-1}$, which suggests that a single qubit on the device is being randomly measured on average every $T_2 = 185ns$. T_2 is also known as the dephasing time of a qubit. Note that the values of $h_{z,\max}$ are the same in Supplementary Figures 4 and 2.

Next, in order to substantiate the Landau-Zener scaling shown in the main text, it is important to relate the

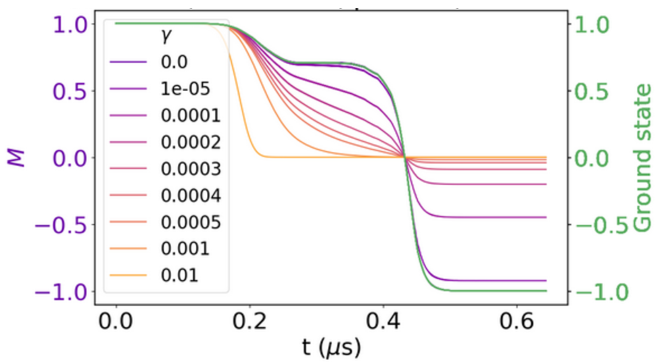

Supplementary Figure 3. Lindblad emulation of single-spin magnetization $M(t)$, including the initial and measurement ramps. Different rates of coupling to the environment γ are indicated in the legend. The green curve with its own vertical axis on the right represents the instantaneous ground state of a single spin throughout the evolution, i.e., at different values of the fields $h_x(t)$ and $h_z(t)$. When $\gamma = 0$, the Lindblad evolution closely follows that of the instantaneous ground state. When γ increases, the curves increasingly collapse to the horizontal line $M = 0$, mimicking the behavior of the device in Supplementary Figure 2.

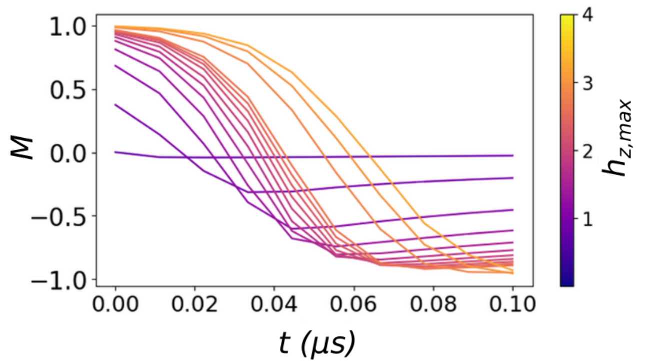

Supplementary Figure 4. Lindblad emulation of single-spin magnetization $M(t)$, without including the initial and measurement ramps in order to be directly comparable to the single-spin quantum simulation results shown in Supplementary Figure 2. Color bar represents different $h_{z,\max}$ values used in the emulation and they are exactly the same as the ones used in the quantum simulation measurements.

rate of change of $M(t)$ with the magnitude of $h_z(t)$. Supplementary Figure 5 shows the time it takes for M in Supplementary Figure 2 to drop from the initial value of 1 down to 0 as we change the magnitude of $h_z(t)$. The scaling is clearly quadratic, $t_{M=0} \propto |h_{z,\max}|^2$.

Finally, we note the large standard deviation of M visible in Supplementary Figure 2. The larger $h_{z,\max}$, the smaller the standard deviation, which suggests that the dynamics are indeed dominated mostly by changes in $h_z(t)$. However, when $h_{z,\max}$ is reduced below 1, the deviation quickly tends to 1, which is of the same order as the measured M at $pt = 0$ and $100ns$. This leads us to conclude that there is a large deviation in the value of

h_z that is imposed on each spin on the actual device. We find that the saturation point of M at $100ns$ in Lindblad emulation depends greatly on the value of h_z and can easily explain the large measured standard deviation.

IV. NUMERICAL EMULATION OF MAGNETIZATION DYNAMICS ON THE D-WAVE QUANTUM ANNEALER

In this section we provide more details of the numerical simulations of the magnetization dynamics, in particular comparing the results of the Bloch-Redfield emulation (discussed in the Methods section of the main text) with the coherent emulation. Our goal is to understand the behavior of the quantum device by employing both closed and open system dynamics emulation. Physically, this discrepancy in the behavior could arise from various factors, such as imperfections in the device's components, environmental noise, or interactions with the surrounding system. First, we find that coherent dynamics do not permit the full decay of the false vacuum due to the conservation of the initial energy determined by the initial false vacuum state, which is clear from the saturation of M far away from the true vacuum value -1. It is only through thermalization dynamics, as shown in our Bloch-Redfield emulations, that the true vacuum can be reached given enough time, as we observe in our quantum simulations. Second, when comparing the rate of false vacuum decay dynamics in coherent and Bloch-Redfield emulations, we find that it is mostly determined by h_x and not by the thermalization rate due to their similar initial behavior. Also, the values of h_x in both the single and multi-spin cases need to be larger than the ones claimed to be used by the device across all emulations. Similarly, h_z is the main driving factor in the case of coherent, Bloch-Redfield, and Lindblad emulation, further supporting the claim that thermalization dynamics are not the dominant effect. Finally, even though both coherent and Lindblad emulations in the single-spin case suggest that an environmental measurement effect is required to reproduce the device's behavior, Bloch-Redfield emulation shows that it is not necessary to reproduce the many-body system dynamics, most likely due to the small h_x values compared to J compared to the single-spin case.

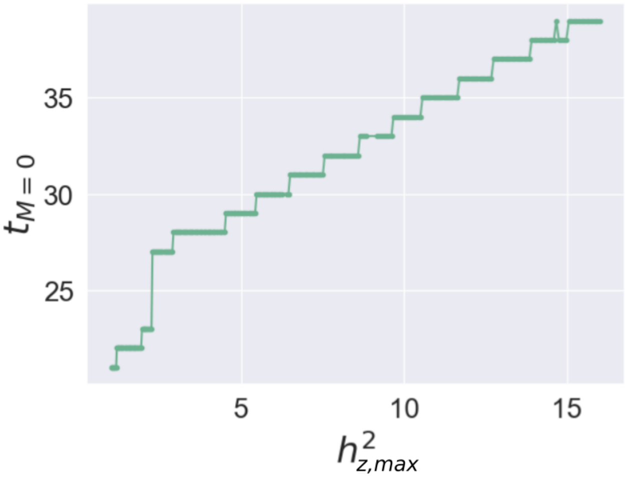

Supplementary Figure 5. The time $t_{M=0}$ it takes for $M(t)$ in Supplementary Figure 2 to reach 0, plotted as a function of $h_{z,\max}$ magnitude. The horizontal axis was squared to show the collapse of $t_{M=0}$ onto a linear curve, suggesting that $t_{M=0} \propto |h_{z,\max}|^2$. The jumps in $t_{M=0}$ are due to the software and hardware filtering that occurs after a user specifies the desired $h_z(t)$.

A. Bloch-Redfield emulation

Supplementary Figure 6 shows measurements on the D-wave device using a 5564-qubit chain with periodic boundary conditions, which are directly compared to its Bloch-Redfield emulation of a 3-qubit ring. The small system that we emulate here exhibits pronounced finite-size effects, however, the location of dynamical resonances is reasonably well-captured even in such small systems and therefore we believe this analysis is useful to shed light on the behavior of the device.

The most important thing to note from the comparison between emulation and experiment is the fact that an order of magnitude larger $h_x \sim 0.015$ values are required to see any kind of dynamics on the relevant timescales $\sim 1\mu s$ compared to the user-specified $h_x \sim 0.002$. This deviation must be kept in mind when translating a user-specified h_x onto the device. The official documentation from D-Wave [1] states that small deviations in $s(t)$ can occur from qubit to qubit, which in our small h_x regime can easily lead to an order of magnitude error in $h_x = A(s)/B(s)$. This suggests additional complexity in modeling of the quantum annealer by having to experimentally extract the h_x inhomogeneity and incorporate it into the emulation of the device. However, this goes beyond the scope of the present study.

Next, we turn to the characteristic shapes of $M(t)$ at different $h_{z,\max}$ magnitudes. All curves initially depart from the value 1 at different times, which is consistent with our emulation results. This means that $h_z(t)$ enforces more and more adiabatic dynamics as its magnitude $h_{z,\max}$ is increased. In Supplementary Figure 6, we also observe modulations in $M(t)$, which track the modulation of the experimental $h_z(t)$ curve. The same effect is observed also in emulation, particularly in the $h_z = -2$ and $h_z = -1$ cases.

However, not all features in $M(t)$ curves in Supplementary Figure 6 can be explained by the emulation. For example, in the annealer data, there are two large oscillations of $M(t)$ at $h_z = 4$, which does not occur in the emulation (similar features can be found at other values of h_z). These oscillations align well with the modulation of the experimental $h_z(t)$, but their origin remains

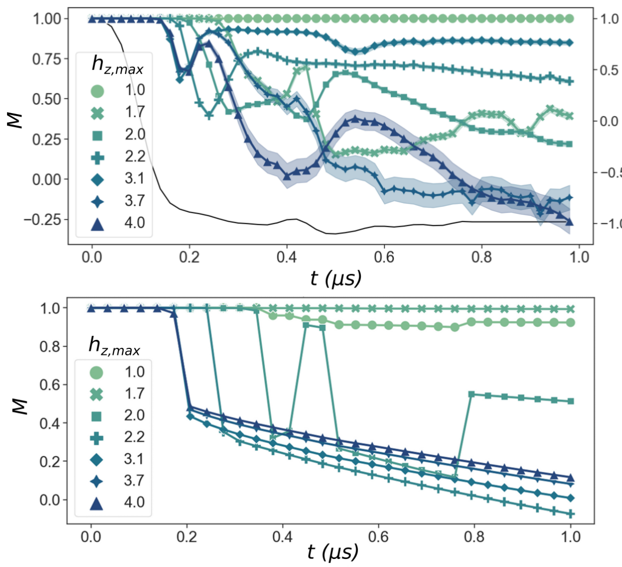

Supplementary Figure 6. Comparison of the measured magnetization on the D-wave device (top) with its Bloch-Redfield emulation (bottom). Top: Magnetization for 5564 qubits was measured at different times for a fixed $h_{x,\max} = 0.002$ and several $h_{z,\max}$ magnitudes indicated in the legend. The shaded area around each curve in the top panel represents the standard deviation obtained during the sampling process on the quantum annealer. The experimentally obtained $h_z(t)$ with magnitude 1 is shown by the black curve with its own axis on the right. Bottom: The Bloch-Redfield emulation for 3 spins on a ring, $h_{x,\max} = 0.015$, and coupling to the environment $\eta = 0.1$ (see the Methods section of the main text). We keep the profile and magnitude of $h_z(t)$ the same as in the quantum simulation.

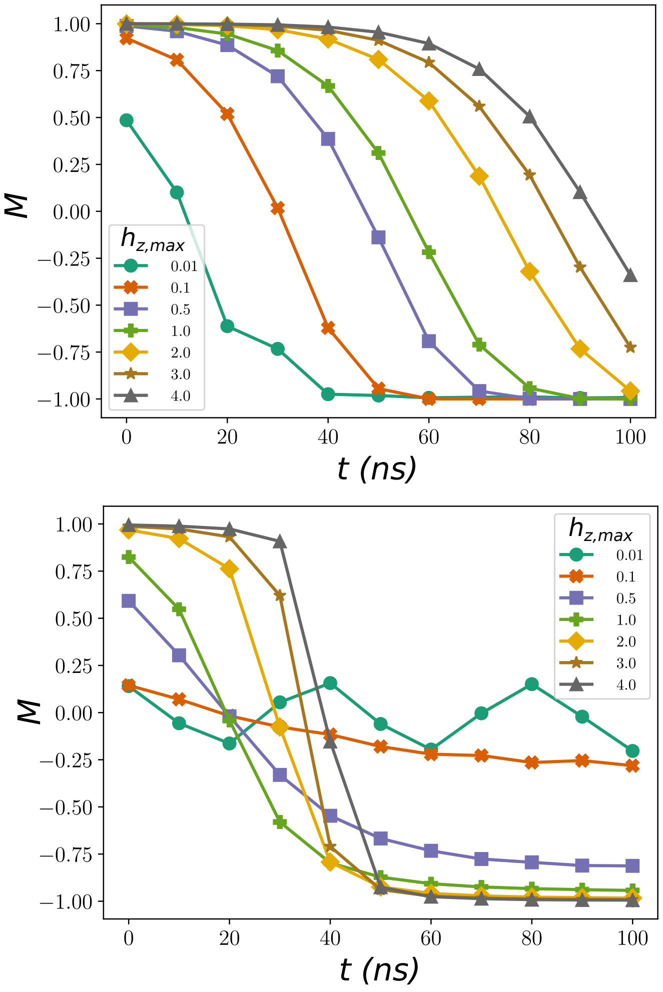

Supplementary Figure 7. Top: Coherent emulation of the single-spin magnetization M (i.e., $J = 0$), with $h_x(t)$ and $h_z(t)$ profiles the same as in Fig. 1 of the main text. Other parameters are $h_{x,\max} = 0.1$, $irt = 100\,\text{ns}$, $pt \in [0, 100]\,\text{ns}$, $mt = 273ns$. Different curves correspond to different values of $h_{z,\max}$. Bottom: Coherent single-spin magnetization M measurements (i.e., $J = 0$), with $h_x(t)$ and $h_z(t)$ fields' schedules as in our quantum simulations, $h_{x,\max} = 0.31$, $irt = 100\,\text{ns}$, $pt \in [0, 100]\,\text{ns}$, $mt = 80\,\text{ns}$, for different values of $h_{z,\max}$. For $h_{z,\max} > 1$, we adjust the modulation according to $h_z(t) \to h_z(t')$, $t' = t/(1 + t_{M0}/200)$ – see text for details.

beyond the scope of this study. Possible explanations include the magnetization of the qubit environment and the consequent feedback response that influences the magnetization of the qubit; the inhomogeneity of h_x; and the deviation of the user-specified $h_z(t)$ magnitude value from the actual experimentally implemented value by a few percent, as stated by the D-Wave documentation.

B. Coherent emulation

Our coherent emulations are based on numerically solving the time-dependent Schrödinger equation, $i\frac{\partial}{\partial t}|\psi(t)\rangle = \hat{H}(t)|\psi(t)\rangle$ to simulate the ideal time-evolution process in the case of a closed system. We work in units $\hbar = 1$ and use a general Hamiltonian $\hat{H}(t)$ that describes a range of quantum annealers and quantum simulators:

$$\hat{H}(t) = \sum_{i=0}^{N-1} (f_i^x(t) h_i^x \sigma_i^x + f_i^y(t) h_i^y \sigma_i^y + f_i^z(t) h_i^z \sigma_i^z) + \sum_{i<j} (F_{ij}^x(t) J_{ij}^x \sigma_i^x \sigma_j^x + F_{ij}^y(t) J_{ij}^y \sigma_i^y \sigma_j^y + F_{ij}^z(t) J_{ij}^z \sigma_i^z \sigma_j^z) \,. \tag{5}$$

The numerical method we use is the Suzuki-Trotter product-formula algorithm [2–6] which is an algorithm for full state vector emulation. The particular implementation of our emulator allows for a decomposition either

into single- and two-qubit terms, or into σ^x-, σ^y- and σ^z-terms. It uses OpenACC so that it runs on GPUs. Due to the exponential growths of the memory requirement to store the state vector, it becomes necessary to use distributed memory of several GPUs, for instance on a supercomputer, if the system size becomes large ($\gtrsim 30$ qubits, depending on the memory of the GPU). For the communication between the GPUs, we use CUDA-aware MPI (Message Passing Interface) following the communication scheme that is also implemented in the Jülich Universal Quantum Computer Simulator (JUQCS) [7–9].

We first focus on the simpler 1-spin case where there are no interactions between the qubits. Based on the dynamics of the fields $h_x(t)$ and $h_z(t)$ used in our quantum simulations in the main text, we would have expected the system to saturate at $M = -1$ because the ground state of the system changes from spin up ($M = 1$) to spin down ($M = -1$) when $h_z(t)$ flips its sign. This is indeed confirmed in the numerics presented in the top panel of Supplementary Figure 7. However, the same thing does not happen on the device, as seen previously in Supplementary Figure 2, where it was observed that the system saturates at values of M greater than -1 when the magnitude of h_z is between 0 and 1.

Using coherent quantum emulations, we have achieved behavior close to that of the annealer, as shown in the bottom panel of Supplementary Figure 7. We make two key modifications to the input fields: (i) we ensure that $h_x(t)$ does not return to zero after the pause time; (ii) we set the measurement ramp time to zero, resulting in an instantaneous measurement of M. After fixing the value of $h_{x,\max}$, we pick the measurement ramp time for which in the coherent emulation M reaches the value 0 after $pt = 20\,$ns, as in Supplementary Figure 2. For example, for $h_{x,\max} = 0.1$, the measurement ramp time should be $mt \approx 80ns$. After fixing the measurement ramp time, we further tune the $h_{x,\max}$ value in the $h_x(t)$ schedule, taking into account that the input values of h_x are highly sensitive to errors in $s(t)$: a 1% deviation in s results in approximately a 10% deviation in h_x. In our example $h_{x,\max} = 0.1$, we observed the behavior similar to the real device with $h_{x,\max} \approx 0.31$. For this value, $s_{\min} \approx 0.376$, which is approximately 17% smaller than the input value. With this value of $h_{x,\max}$ and the ramp time estimated previously, we proceeded to emulate the scenario depicted in Supplementary Figure 2. We also took into account the delay in the $h_z(t)$ quench by adjusting $h_z(t)$ as follows: for $h_{z,\max} > 1$, $h_z(t) \to h_z(t')$, $t' = t/(1 + t_{M0}/200)$, where the value t_{M0} is obtained by the relation $t_{M0}(h_z^2)$ showed in Supplementary Figure 5. The result is shown in the bottom panel of Supplementary Figure 7.

After approximately reproducing the single-qubit dynamics, we investigated the case of coupled $N = 10$ qubits. Comparison of the modified coherent emulation, which takes into account the single-qubit results, with the real device is shown in Supplementary Figure 8. Based on the previous discussion of the uncoupled case, we used

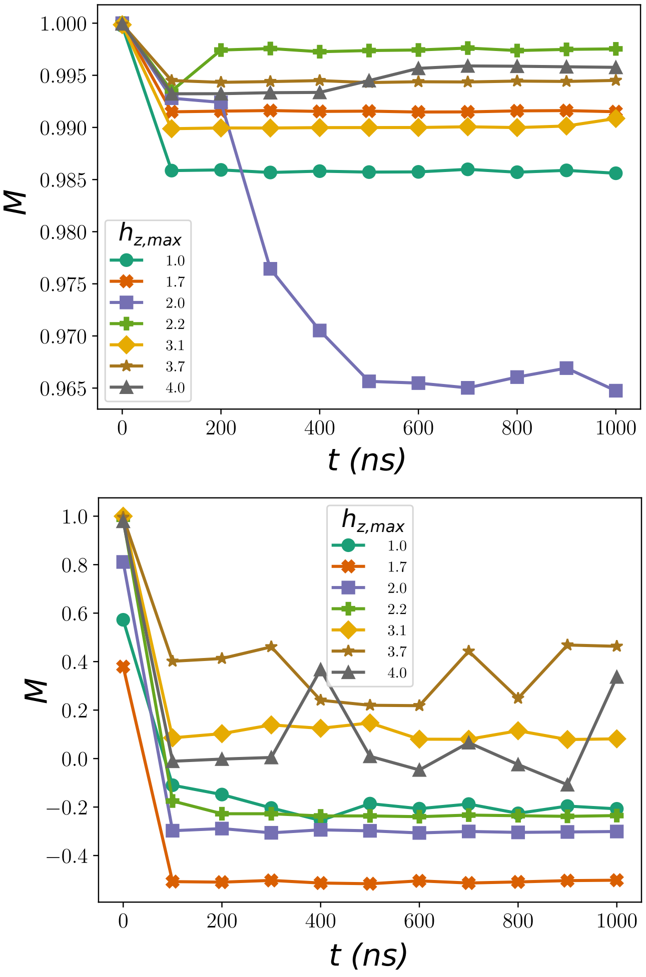

Supplementary Figure 8. Coherent emulation of the magnetization M for $N = 10$ spins (i.e., the coupling is set to $J = 1$), with $h_x(t)$ and $h_z(t)$ fields' schedules as in Supplementary Figure 7, with $h_{x,\max} = 0.002$ (top panel) and $h_{x,\max} = 0.029$ (bottom panel). Other parameters are $irt = 100ns$, $pt \in [0, 100]$ ns, $mt = 80ns$, for different values of $h_{z,\max}$. For $h_{z,\max} > 1$, we adjust the modulation according to $h_z(t) \to h_z(t')$, $t' = t/(1 + t_{M0}/200)$ – see text for details.

both the s_{h_x} value given as input to the device and a value that is 17% smaller, resulting in $s_{h_x,\text{eff}} \approx 0.527$. In Supplementary Figure 8 we tested both values of $h_{x,\max} = 0.002$ and 0.029, up to $1\,\mu$s and for different $h_{z,\max}$ values.

We observe significant differences between the behavior of the real device, depicted in Supplementary Figure 6, and the coherent emulations shown in Supplementary Figure 8. In the coherent case, there is a rapid decline in M across all $h_{z,\max}$ values, particularly noticeable for $h_{z,\max} = 1$, that does not occur on the device. Furthermore, following the drop in M in the coherent case, the system tends to saturate to the attained value of M for most $h_{z,\max}$ values. However, in the real device, oscillations in M are present, and the decline is more grad-

ual. Nevertheless, it is worth noting that the expected resonances were still evident in the coherent emulations, indicating a fundamental consistency in the underlying dynamics.

Several factors may contribute to the disparity between coherent simulations and the real device behavior, such as thermalization and environment-induced measurement, inhomogeneity across different qubits in terms of h_x and h_z, as well as the system size limitation of the coherent emulation to only 10 qubits, while the dynamics of the real device involved 5564 qubits.

V. EFFECTIVE MODELS AT DIFFERENT RESONANCES

The goal of this section is to derive effective Hamiltonians that approximately describe the dynamics of the Ising model in Eq. (1) in the regime $h_z, J \gg h_x$ (and with some extra conditions between h_z and J, to be specified below). In this limit, the classical energy given by $\hat{\sigma}^z$ terms is the dominant scale and any process that changes it is highly suppressed. Therefore, the only allowed processes are those that conserve this classical energy, which can only happen at specific resonances between h_z and J. In these cases, we will use a Schrieffer-Wolff transformation [10] to derive the effective Hamiltonian at leading order. All our derivations will be restricted to the Hilbert space sector containing the all-up state $|\psi_0\rangle = |\uparrow\uparrow\uparrow\ldots\uparrow\rangle$, i.e., one of our vacuum states.

Let us consider starting from the state $|\uparrow\uparrow\uparrow\ldots\uparrow\rangle$ and flipping n neighboring spins:

$$\cdots\uparrow\uparrow\underbrace{\downarrow\downarrow\cdots\downarrow\downarrow}_{n}\uparrow\uparrow\cdots\,. \tag{6}$$

This creates two domain walls ($\uparrow\downarrow$ and $\downarrow\uparrow$) at the edges of the down-spin domain, and thus an energy increase of $+4J$. At the same time, n spins being down leads to an energy change of $+2nh_z$. In order for this process to be resonant, we require $4J + 2nh_z = 0$, implying the resonance condition

$$h_z = -2J/n, \tag{7}$$

with $n \geq 1$ being an integer.

The resonance condition in Eq. (7) was previously obtained in Ref. [11], but for the sector containing the state $|\downarrow\downarrow\downarrow\ldots\downarrow\rangle$, hence their version of Eq. (7) is related to ours via a simple sign flip. Alternatively, we can notice that our state of interest and that of Ref. [11] are related by the action of $\hat{X} = \prod_j \hat{\sigma}^x_j$. Applying the transformation $\hat{X}$ to the Hamiltonian (1) only changes the sign in front of h_z term. However, in contrast to Ref. [11], we are not only interested in the creation of bubbles of n consecutive $\downarrow$-spins, but also in the effective dynamics. Thus, we will go beyond the effective description in Ref. [11] and derive the effective Schrieffer-Wolff terms at various orders.

Importantly, as $\hat{\sigma}^x$ only flips a single spin at a time, the resonant process at $h_z = -2J/n$ will only appear at n-th order and so will be suppressed by a prefactor of $\approx h_x^n/J^{n-1}$. In addition, there will always be diagonal contributions at second order corresponding to flipping a spin down and flipping it back up. These will be much stronger than bubble creation for any $n > 2$, and thus we mainly focus on the cases $n = 1$ and $n = 2$ where there are dynamical processes at leading order. These will be discussed in Sections V A and V B respectively. Finally, in Section V C we will study the generic n case and show that it is fairly similar to $n = 2$.

In writing down the effective Hamiltonians below, we will make use of the projectors

$$\hat{P}^{\uparrow} = |\uparrow\rangle\langle\uparrow| = \frac{1+\hat{\sigma}^z}{2}, \quad \hat{P}^{\downarrow} = |\downarrow\rangle\langle\downarrow| = \frac{1-\hat{\sigma}^z}{2}, \tag{8}$$

as well as the standard Pauli raising and lowering operators, $\hat{\sigma}^{\pm} \equiv (\hat{\sigma}^x \pm i\hat{\sigma}^y)/2$. To demonstrate the accuracy of the effective Hamiltonian description, we will compare several dynamical quantities in the full model against the effective description. The quantities we will study include the expectation values of spin magnetization, Eq. (2), and the return fidelity of the wave function after a quench, $\mathcal{F}(t) = |\langle\psi_0|\psi(t)\rangle|^2$, where we consider a closed system and the time evolved state is $|\psi(t)\rangle = e^{-i\hat{H}t}|\psi_0\rangle$. Unless specified otherwise, we will work in units $J = 1$ throughout this section.

A. $h_z = -2J$ resonance: the PXP model

We first focus on the $n = 1$ resonance at $h_z = -2J$. As discussed above, the leading Hamiltonian is at first order, since creating a single down-spin leads to two new domain walls and so this incurs an energy $+4J$ from the Ising term but $+2h_z = -4J$ from the field term. As such, creating isolated down spins is resonant. This is not true for neighboring down spins, as this does not increase the number of domain walls. The resulting Hamiltonian is

$$\hat{H}^{(1)}_{\text{eff}} = -h_x \sum_{j=1}^{N} \hat{P}^{\uparrow}_{j-1}\hat{\sigma}^x_j\hat{P}^{\uparrow}_{j+1}. \tag{9}$$

Up to the exchange of up and down spins, we recognize this as the PXP model Hamiltonian [12, 13], which hosts quantum many-body scars [14, 15]. The PXP model arises in a different context as the effective model in a 1D chain of Rydberg atoms in the "Rydberg blockade" regime, where strong interactions between neighboring atoms prevent them from being simultaneously excited.

The effective model (9) has a particularly simple constrained Hilbert space: there are no processes that can create neighboring down-spins. At the level of the full Hilbert space, one can thus rely on the projector $\hat{P}^{\downarrow}_j\hat{P}^{\downarrow}_{j+1}$ to detect if the wave function is escaping the constrained sector. This can be diagnosed by computing

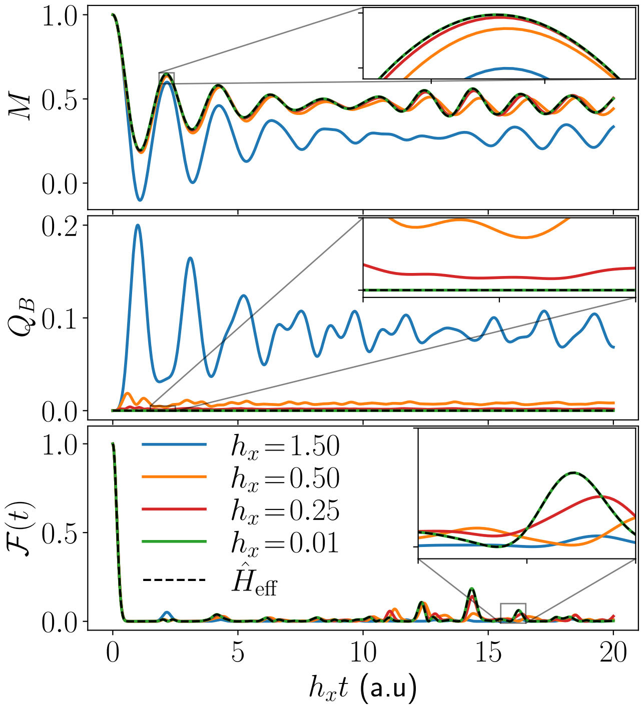

Supplementary Figure 9. Dynamics in the Ising model (1) at $h_z = -2$ resonance. All data is for $N = 20$ spins on a ring with $J = 1$. We perform a global quench by preparing the system in the $|\uparrow\uparrow\ldots\uparrow\rangle$ state and letting it evolve under unitary dynamics. We characterize the dynamics by measuring the magnetization, blockade violation Q_B in Eq. (10), and fidelity. The prediction of the effective model in Eq. (9), shown by dashed line, accurately approximates the dynamics for $h_x \leq 0.25$.

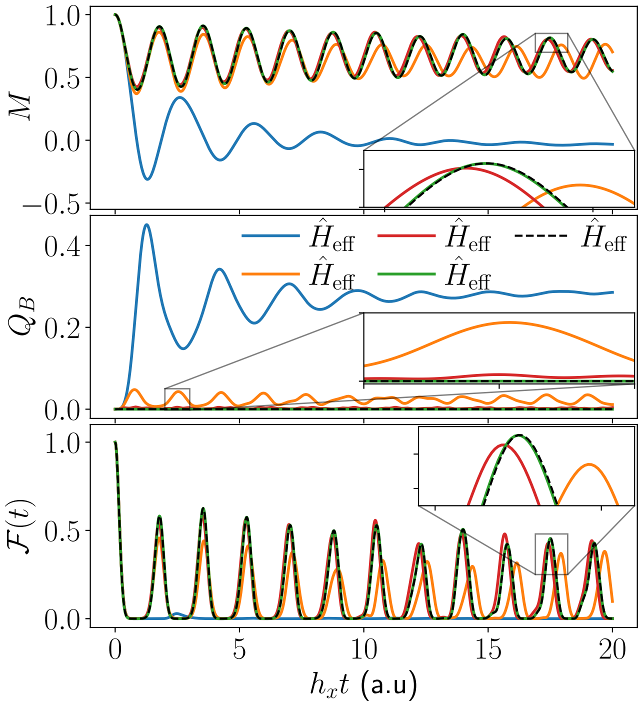

Supplementary Figure 10. Dynamics in the Ising model (1) weakly detuned away from the resonance at $h_z = -2J + 0.84h_x$. All data is for $N = 20$ spins on a ring with $J = 1$. We perform a global quench by preparing the system in the $|\uparrow\uparrow\ldots\uparrow\rangle$ state and letting it evolve under unitary dynamics. We characterize the dynamics by measuring the magnetization, blockade violation Q_B in Eq. (10), and fidelity. The prediction of the effective model in Eq. (9), shown by dashed line, accurately approximates the dynamics for $h_x \leq 0.25$. Unlike Supplementary Figure 9, the dynamics here exhibits regular oscillations, which is a signature of quantum many-body scars [16].

$\prod_j(1-\hat{P}^{\downarrow}_j\hat{P}^{\downarrow}_{j+1})$, which should be equal to 1 if the dynamics obeys the constraint. However, this operator is global and thus difficult to measure. Instead, we can rely on the local operator

$$\hat{Q}_B = \frac{1}{N}\sum_j \hat{P}^{\downarrow}_j\hat{P}^{\downarrow}_{j+1} = \frac{1}{4} + \frac{1}{4N}\sum_j \hat{\sigma}^z_j\hat{\sigma}^z_{j+1} - \frac{1}{2N}\sum_j \hat{\sigma}^z_j. \tag{10}$$

The expectation value $Q_B = \langle\hat{Q}_B\rangle$ should be ≈ 0 if the blockade is approximately obeyed and ≈ 1 if not. Supplementary Figure 9 illustrates the accuracy of the effective model (9) in describing the full quantum dynamics as long as the field $h_x \lesssim 0.25$. In the same range, we also see that the blockade condition (10) is well-obeyed even at late times.

In quantum simulations, it is important to know how the effective description gets modified slightly *off* resonance, i.e., when there is a small mismatch between $-2J$ and h_z. Let us set $h_z = -2J + \delta$ or, equivalently, introduce the detuning $\delta = h_z + 2J$. Then adding a single down-spin costs energy $4J + 2h_z = 2\delta$, creating an effective detuning. As long as $\delta \gg |J|$, we can incorporate the detuning as a perturbation to the classical Hamiltonian. The Hilbert space sector remains the same but the effective Hamiltonian is now

$$\begin{aligned}\hat{H}^{(1)}_{\rm eff}(\delta) &= -h_x\sum_{j=1}^{N}\hat{P}^{\uparrow}_{j-1}\hat{\sigma}^x_j\hat{P}^{\uparrow}_{j+1} - \delta\sum_{j=1}^{N}\hat{\sigma}^z_j \\ &= -h_x\sum_{j=1}^{N}\hat{P}^{\uparrow}_{j-1}\hat{\sigma}^x_j\hat{P}^{\uparrow}_{j+1} + 2\delta\sum_{j=1}^{N}\hat{P}^{\downarrow}_j,\end{aligned} \tag{11}$$

where we have dropped the irrelevant constant term $-2N\delta$ in the final expression. The resulting effective model is seen to be the same as the PXP model in the presence of chemical potential, as δ yields a simple energy penalty for creating excitations (spin flips) [14].

The PXP model with the chemical potential, Eq. (11), has recently been shown to exhibit quantum many-body scarring from one of our vacuum states, $|\uparrow\uparrow\ldots\uparrow\rangle$ [16]. The optimal value of detuning that leads to scarring was found to be $\approx \pm 1.68$ times the dynamical term, hence we expect quantum revivals around $\delta = \pm 1.68h_x/2 = \pm 0.84h_x$ (or equivalently $h_z = -2J \pm 0.84h_x$). This is il-

lustrated in Supplementary Figure 10, where we see regular oscillations in the magnetization and fidelity. This is in sharp contrast with Supplementary Figure 9, where scarring is not expected from the vacuum state [14]. Thus, our quantum annealer platform that realizes the model (1) allows to directly probe quantum many-body scars in the same dynamical regimes in which we studied the bubble formation in the main text.

We have also computed higher order corrections to the effective Hamiltonian. For example, at second order we obtain

$$\hat{H}^{(2)} = \frac{h_x^2}{4J}\Big[\sum_{j=1}^{N}\hat{P}^{\uparrow}_{j-1}(\hat{\sigma}^+_j\hat{\sigma}^-_{j+1}+\hat{\sigma}^-_j\hat{\sigma}^+_{j+1})\hat{P}^{\uparrow}_{j+2} + 2\sum_{j=1}^{N}\hat{P}^{\downarrow}_j - \frac{3}{2}\sum_{j=1}^{N}\hat{P}^{\downarrow}_{j-1}\hat{P}^{\downarrow}_{j+1}\Big], \tag{12}$$

which is independent of δ. Moreover, at fourth order, there will be terms proportional to $h_x^3/(2J)^2$ and others proportional to $\delta h_x^2/(2J)^2$.

It is important to note that none of these terms – or even higher order ones – can lead to the creation of consecutive down-spins from the state $|\uparrow\cdots\uparrow\rangle$. Indeed, such configurations would lead to a net energy difference from the $|\uparrow\cdots\uparrow\rangle$ state, as they all have a different classical energy than this state. Such transitions between $|\uparrow\cdots\uparrow\rangle$ and states with neighboring down-spins are thus highly suppressed.

However, if we instead initialize the system in a configuration with neighboring down-spins, then we have to consider an additional term in the second-order processes:

$$\hat{H}^{(2)}_{\rm int} = -\frac{h_x^2}{8J}\Big[\sum_{j=1}^{N}\hat{P}^{\downarrow}_{j-1}(\hat{\sigma}^+_j\hat{\sigma}^-_{j+1}+\hat{\sigma}^-_j\hat{\sigma}^+_{j+1})\hat{P}^{\downarrow}_{j+2}\Big]. \tag{13}$$

This terms allows down-spins to move from one domain to a neighboring one. The only restriction is that none of the domains vanish because of this. So this cannot happen if all domains are of size 1, as none of them can give a down-spin to the other. It is thus irrelevant for the dynamics we consider from the $|\uparrow\cdots\uparrow\rangle$ state. However, in other sectors it means that neighboring down-spins are not completely frozen. In conjunction with isolated down-spins being able to move on their own, this greatly increases the mobility of down-spins.

B. $h_z = -J$ resonance

The case $h_z = -J$ is more complicated as there is no resonant term at first order. Indeed, as there are no boundaries to the chain there is no way to create a single domain wall – their number only changes in steps of two. In order to compensate for the creation of two domain walls, we thus require two spins to be $\downarrow$, which cannot be done in a single move. We emphasize that this is only true for PBCs. With open boundary conditions, the spins at each boundary can resonantly flip, while the bulk of the chain cannot. Thus, we would get terms $h_x\hat{\sigma}^x_1$ and $h_x\hat{\sigma}^x_N$ at first order. In the rest of this section, we focus on the PBC case.

The leading order of the effective Hamiltonian is at second order and reads:

$$\hat{H}^{(2)}_{\rm eff} = \frac{h_x^2}{J}\sum_{j=1}^{N}\big[\frac{2}{3}\hat{P}^{\downarrow}_{j-1}\hat{\sigma}^z_j\hat{P}^{\downarrow}_{j+1} - \frac{1}{2}\hat{\sigma}^z_{j-1}\hat{\sigma}^z_j\hat{\sigma}^z_{j+1}\big] + \frac{h_x^2}{J}\sum_{j=1}^{N}\hat{P}^{\uparrow}_{j-1}\left(\hat{\sigma}^+_j\hat{\sigma}^-_{j+1}+\hat{\sigma}^-_j\hat{\sigma}^+_{j+1}-\hat{\sigma}^+_j\hat{\sigma}^+_{j+1}-\hat{\sigma}^-_j\hat{\sigma}^-_{j+1}\right)\hat{P}^{\uparrow}_{j+2} - \frac{h_x^2}{3J}\sum_{j=1}^{N}\hat{P}^{\downarrow}_{j-1}(\hat{\sigma}^+_j\hat{\sigma}^-_{j+1}+\hat{\sigma}^-_j\hat{\sigma}^+_{j+1})\hat{P}^{\downarrow}_{j+2}. \tag{14}$$

One can notice that the second line can also be written compactly as $\hat{P}^{\uparrow}_{j-1}\hat{\sigma}^y_j\hat{\sigma}^y_{j+1}\hat{P}^{\uparrow}_{j+2}$.

Let us briefly discuss the different terms in the effective Hamiltonian (14). The XY terms, $\hat{\sigma}^+_j\hat{\sigma}^-_{j+1}+\hat{\sigma}^-_j\hat{\sigma}^+_{j+1}$, dressed with projectors, move a spin from one domain to another, meaning they effectively move a domain wall. This does not change the number of $\downarrow$ spins or the number of domain walls and so is resonant. The dressed $\hat{P}^{\uparrow}_{j-1}\left(\hat{\sigma}^+_j\hat{\sigma}^+_{j+1}+\hat{\sigma}^-_j\hat{\sigma}^-_{j+1}\right)\hat{P}^{\uparrow}_{j+2}$ terms create two down spins in a domain of $\uparrow$ spins. This creates two domain walls, compensating the energy loss from the new $\downarrow$-spins. Finally, the diagonal term corresponds to flipping a spin and immediately flipping it back.

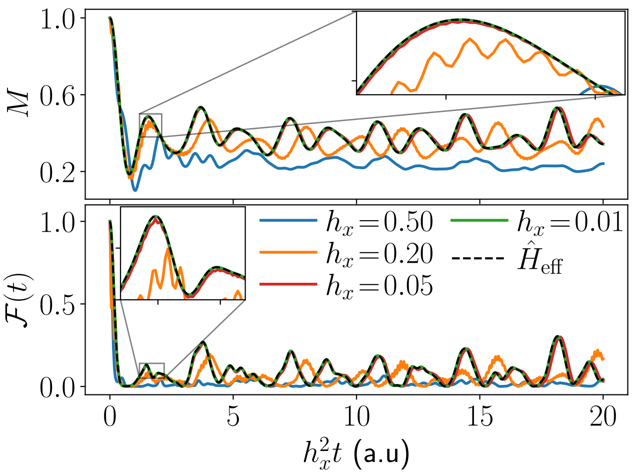

Supplementary Figure 11. Dynamics in the Ising model (1) at $h_z = -J$ resonance. All data is for $N = 20$ spins on a ring with $J = 1$. We perform a global quench by preparing the system in the $|\uparrow\uparrow\ldots\uparrow\rangle$ state and letting it evolve under unitary dynamics. We characterize the dynamics by measuring the magnetization and fidelity. The prediction of the effective model in Eq. (14), shown by dashed line, gives a good approximation of the dynamics for $h_x \leq 0.05$.

Supplementary Figure 11 shows that the effective model (14) agrees well with the full dynamics for small

values of $h_x \lesssim 0.05$. It also shows how rescaling time by h_x^2 makes the different quenches align well, illustrating that the overall prefactor dictating the timescale is J/h_x^2.

The Hilbert space structure of the effective model (14) is more complicated, as there is no obvious local quantity that is the same for all states in the relevant Hilbert space sector. Indeed, while domains of two ↓-spins are initially created, the domain walls can move thanks to the dressed XY terms. This means than different bubbles can exchange ↓-spins and change their size in doing so. Nevertheless, one can notice that the model preserves the parity of the total number of ↑-spins, as all terms change this number by zero or two.

Unlike the $h_z = -2J$ resonance, where the effective PXP model had been independently studied in the context of Rydberg atoms [14, 17] and tilted optical lattices [16], to the best of our knowledge, there have been no investigations of the effective model at $h_z = -J$ resonance in Eq. (14). Our preliminary studies of the level statistics suggest that the model is chaotic (data not shown), and it would be interesting to explore the possibility of weak ergodicity breaking in it. Similar to the PXP case in Eq. (11), this can be probed by introducing slight detuning δ, with $h_z = -J + \delta$. The detuning, once again, can be included as a perturbation to the classical Hamiltonian, but here there is a non-zero effective Hamiltonian at first order:

$$\hat{H}^{(1)}_{\text{eff}}(\delta) = -\delta \sum_j \hat{\sigma}^z_j. \tag{15}$$

The second order terms are unchanged, but there are now non-zero terms at third order with strength $\delta h_x^2/J^2$.

C. $n > 2$ resonances

For $n > 2$ resonances, the strength of the process creating n consecutive down-spins in the state $|\uparrow\uparrow\cdots\uparrow\rangle$ is proportional to h_x^n. The exact prefactor can be computed by accounting for all possible paths between the initial and final states. For example, for $n = 3$ it was found in Ref. [11] that the prefactor is $-81/64$. On top of the bubble creation process, we can ask if these bubbles can then move around or interact. The former was found at second order for $n = 1$ in Sec. V A, while the latter was found (also at second order) for $n = 2$ in Sec. V B.

Let us first consider hopping. The exact value of n is actually irrelevant, so we will use $n = 3$ for simplicity. We can try to move a 3-bubble $|\uparrow\downarrow\downarrow\downarrow\uparrow\uparrow\rangle$ to the right into $|\uparrow\uparrow\downarrow\downarrow\downarrow\uparrow\rangle$ by doing two flips. The two processes that can lead to that are

$$|\uparrow\downarrow\downarrow\downarrow\uparrow\uparrow\rangle \rightarrow |\uparrow\downarrow\downarrow\downarrow\downarrow\uparrow\rangle \rightarrow |\uparrow\uparrow\downarrow\downarrow\downarrow\uparrow\rangle\,, \tag{16}$$

$$|\uparrow\downarrow\downarrow\downarrow\uparrow\uparrow\rangle \rightarrow |\uparrow\uparrow\downarrow\downarrow\uparrow\uparrow\rangle \rightarrow |\uparrow\uparrow\downarrow\downarrow\downarrow\uparrow\rangle\,. \tag{17}$$

For either process, the number of domain walls is not changed by the flips. The only change in energy is due to the h_z field term. For the process in Eq. (16) we have $\Delta E = E_0 - E_1 = -2h_z$. For the process in Eq. (17), we have a spin flipped up instead of down. This gives us $\Delta E' = +2h_z$. The total matrix element of the hopping is then

$$h_x^2\left(\frac{1}{\Delta E} + \frac{1}{\Delta E'}\right) = \frac{h_x^2}{2h_z}(-1+1) = 0, \tag{18}$$

so the two processes cancel each other. A similar computation can be done for any $n > 1$, including $n = 2$, meaning that in these cases the bubbles will never be allowed to hop. The case $n = 1$ is different. Indeed, the process equivalent to Eq. (17) is $|\uparrow\downarrow\uparrow\uparrow\rangle \rightarrow |\uparrow\uparrow\uparrow\uparrow\rangle \rightarrow |\uparrow\uparrow\downarrow\uparrow\rangle$, which does not conserve the number of domain walls. This means that the energy of this process will not be the opposite of the other process. In fact, for $n = 1$ this process is actually resonant. Therefore, the only non-resonant process that will contribute to hopping is the one equivalent to Eq. (16) and there can be no cancellation. Thus, there is a non-zero effective term at second order that allows 1-bubble to hop.

The exact opposite happens for interactions between bubbles. For $n = 1$, we cannot remove a down-spin from a bubble without destroying it. Therefore, there cannot be any exchange between bubbles. However, this not true for higher n. Let us consider two bubbles (with the left one of size at least two) and a single site between them: $|\ldots\downarrow\downarrow\uparrow\downarrow\rangle$. Then, there are two processes that can transfer a spin down from the left bubble to the right one:

$$|\downarrow\downarrow\uparrow\downarrow\rangle \rightarrow |\downarrow\downarrow\downarrow\downarrow\rangle \rightarrow |\downarrow\uparrow\downarrow\downarrow\rangle\,, \tag{19}$$

$$|\downarrow\downarrow\uparrow\downarrow\rangle \rightarrow |\downarrow\uparrow\uparrow\downarrow\rangle \rightarrow |\downarrow\uparrow\downarrow\downarrow\rangle\,. \tag{20}$$

The energy differences of these processes are, respectively, $\Delta E = 4J - 2h_z = -2nh_z - 2h_z = -2(1+n)h_z$ and $\Delta E' = 2h_z$. We get a matrix element of strength

$$h_x^2\left(\frac{1}{\Delta E} + \frac{1}{\Delta E'}\right) = \frac{h_x^2}{2h_z}\frac{n}{n+1} = -\frac{h_x^2}{4J}\frac{n^2}{n+1}. \tag{21}$$

In the end, we indeed recover a hopping process proportional to h_x^2/h_z, corresponding to $\hat{P}^{\downarrow}_{j-1}\left(\hat{\sigma}^+_j\hat{\sigma}^-_{j+1} + \hat{\sigma}^-_j\hat{\sigma}^+_{j+1}\right)\hat{P}^{\downarrow}_{j+2}$. For $n = 2$, the strength is $h_x^2/(3h_z)$ or equivalently $-h_x^2/(3J)$, matching with the previous derivation in Eq. (14).

The previous process allows bubble to change size, and in particular a bubble can shrink down to a simple 1-bubble by giving down-spins to other bubbles. The 1-bubbles can then hop according to the following two processes:

$$|\uparrow\downarrow\uparrow\uparrow\rangle \rightarrow |\uparrow\downarrow\downarrow\uparrow\rangle \rightarrow |\uparrow\uparrow\downarrow\uparrow\rangle\,, \tag{22}$$

$$|\uparrow\downarrow\uparrow\uparrow\rangle \rightarrow |\uparrow\uparrow\uparrow\uparrow\rangle \rightarrow |\uparrow\uparrow\downarrow\uparrow\rangle\,. \tag{23}$$

The energy differences of these processes are, respectively, $\Delta E = -2h_z$ and $\Delta E' = 4J + 2h_z = -2(n-1)h_z$. As in the case $n = 1$, the energy differences of the two processes are not opposite since in one of them we change

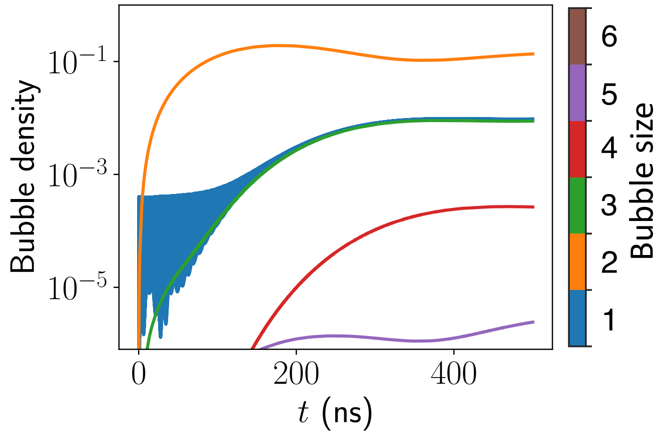

Supplementary Figure 12. Matrix-product state simulation of the dynamics in the Ising model (1) with experimentally relevant parameters at $h_z = -J$ and $h_x = 0.02J$. The system contains $N = 100$ spins and open boundary conditions. We perform a global quench by preparing the system in the $|\uparrow\uparrow\ldots\uparrow\rangle$ state and letting it evolve under unitary dynamics. As expected, 2-bubbles dominate while 1- and 3-bubble are next. The oscillations in 1-bubbles at short times are due to h_x being finite. They can be further suppressed by choosing a lower value of this parameter.

the number of domain walls. The effective matrix element then has the strength

$$h_x^2\left(\frac{1}{\Delta E}+\frac{1}{\Delta E'}\right) = -\frac{h_x^2}{2h_z}\frac{n}{n-1} = \frac{h_x^2}{4J}\frac{n^2}{n-1}. \quad (24)$$

In the end, we recover a hopping process corresponding to $\hat{P}^{\uparrow}_{j-1}\left(\hat{\sigma}^+_j\hat{\sigma}^-_{j+1}+\hat{\sigma}^-_j\hat{\sigma}^+_{j+1}\right)\hat{P}^{\uparrow}_{j+2}$. For $n = 2$, we recover h_x^2/J, as in Eq. (14).

Finally, we can also recognize that there will always be diagonal terms at second order. Indeed, it is always possible to flip a spin in a non-resonant way and then flip it back. This gives us four diagonal terms: $\frac{n}{4J}\frac{\hat{P}^{\downarrow}_{j-1}\hat{\sigma}^z_j\hat{P}^{\downarrow}_{j+1}}{n+1}$, $\frac{n}{4J}\hat{P}^{\downarrow}_{j-1}\hat{\sigma}^z_j\hat{P}^{\uparrow}_{j+1}$, $\frac{n}{4J}\hat{P}^{\uparrow}_{j-1}\hat{\sigma}^z_j\hat{P}^{\downarrow}_{j+1}$ and $-\frac{n}{4J}\frac{\hat{P}^{\uparrow}_{j-1}\hat{\sigma}^z_j\hat{P}^{\uparrow}_{j+1}}{n-1}$.

If we now combine all these terms together, we find that for any $n > 1$, we have the following Hamiltonian at second order:

$$\begin{aligned}\hat{H}^{(2)}_{\text{eff}} = &\frac{h_x^2 n}{4J}\sum_{j=1}^{N}\Big[\frac{\hat{P}^{\downarrow}_{j-1}\hat{\sigma}^z_j\hat{P}^{\downarrow}_{j+1}}{n+1}+\hat{P}^{\uparrow}_{j-1}\hat{\sigma}^z_j\hat{P}^{\downarrow}_{j+1} \\ &\qquad +\hat{P}^{\downarrow}_{j-1}\hat{\sigma}^z_j\hat{P}^{\uparrow}_{j+1}-\frac{\hat{P}^{\uparrow}_{j-1}\hat{\sigma}^z_j\hat{P}^{\uparrow}_{j+1}}{n-1}\Big] \\ &+\frac{h_x^2 n^2}{4J(n-1)}\sum_{j=1}^{N}\hat{P}^{\uparrow}_{j-1}\left(\hat{\sigma}^+_j\hat{\sigma}^-_{j+1}+\hat{\sigma}^-_j\hat{\sigma}^+_{j+1}\right)\hat{P}^{\uparrow}_{j+2} \\ &-\frac{h_x^2 n^2}{4J(n+1)}\sum_{j=1}^{N}\hat{P}^{\downarrow}_{j-1}(\hat{\sigma}^+_j\hat{\sigma}^-_{j+1}+\hat{\sigma}^-_j\hat{\sigma}^+_{j+1})\hat{P}^{\downarrow}_{j+2}.\end{aligned} \quad (25)$$

For $n = 2$, we have to add the process creating 2-bubbles and we then recover Eq. (14). For $n > 2$, Eq. (25) is the leading order Hamiltonian. However, we recognize that the state $|\uparrow\uparrow\cdots\uparrow\rangle$ is an eigenstate of this Hamiltonian with eigenvalue $-N\frac{h_x^2}{4J}\frac{n}{n-1}$. As such, the bubble creation term that occurs at order n is still the leading dynamical term.

For $n > 1$, bubble creation and bubble interactions conspire to create very rich dynamics. In Supplementary Figure 12, we show a large scale matrix-product-state simulation of the full Hamiltonian at the $n = 2$ resonance. While 2-bubbles dominate, as expected, 1- and 3-bubbles appear together from the interactions of 2-bubbles. Larger n-bubbles also appear, but they need $n-1$ different 2-bubbles to be close to each other and interact. As such, the larger the bubbles the more suppressed they are.

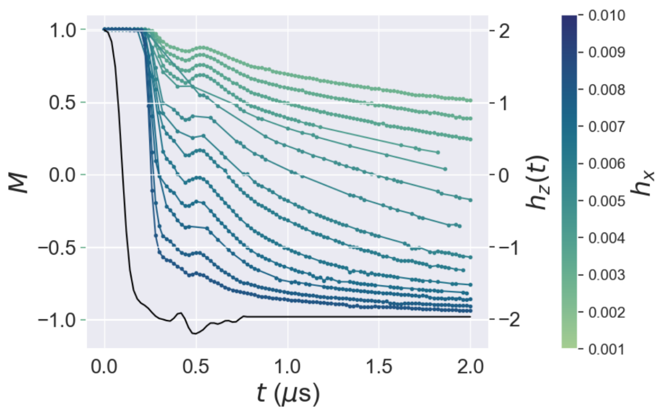

Supplementary Figure 13. Measurements of magnetization M at the 1-bubble resonance $h_z = -2J$ for different values of h_x shown on the color bar. The black curve shows the $h_z(t)$ measured directly on the device with its scale on the right axis of the plot.

VI. QUANTUM SIMULATION OF THE $h_z = -2J$ RESONANCE

Supplementary Figure 13 shows measurements of magnetization [Eq. (2)] taken on the quantum annealer at the 1-bubble resonance $h_z = -2J$ and using the protocol described in Sec. III A. Up to times of approximately $0.75\mu s$, $M(t)$ curves change according to the modulation of $h_z(t)$ with a varying degree of added decay that depends on h_x. Increasing h_x increases the rate of M decay towards the value -1, which is consistent with simply increasing the rate of dynamics. After the modulation of $h_z(t)$ settles, the dynamics change qualitatively, showing only an h_x-dependent decay towards the value -1, which represents the system's ground state.

Supplementary Figure 14 elucidates the two charactertistic timescales in the dynamics of magnetization presented in Supplementary Figure 13: it shows how the two timescales of $M(t)$ collapse onto the same curve when scaling the time axis with h_x or h_x^2. As we discuss in

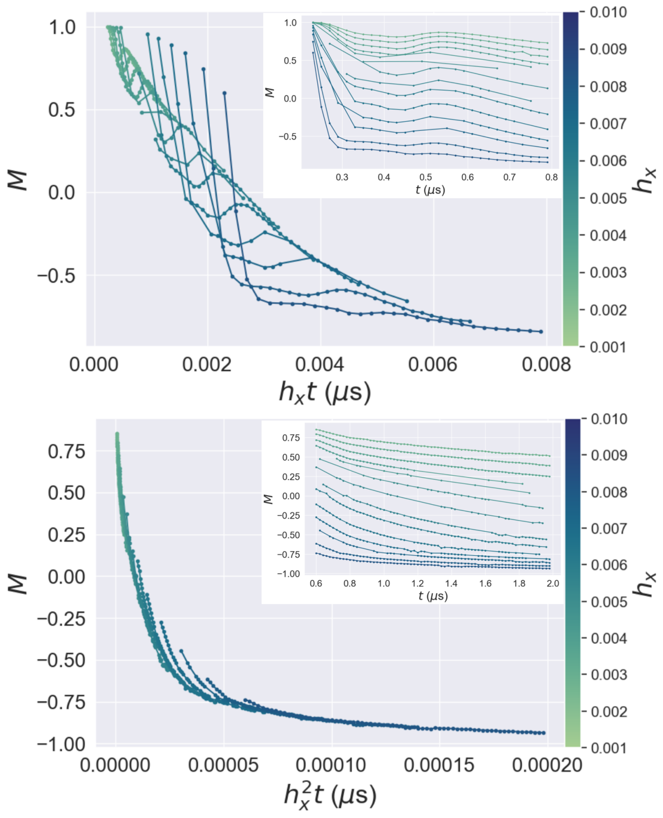

Supplementary Figure 14. Different timescales in the dynamics of magnetization M measured at the 1-bubble resonance $h_z = -2J$ in Supplementary Figure 13. Top: M for $t \lesssim 0.75\mu s$ plotted as function of rescaled time $h_x t$ for different values of h_x shown on the color bar, with the raw data shown in the inset. The curves collapse onto a single curve according to the h_x scaling law, suggesting that the effective Hamiltonian governing the dynamics is proportional to h_x. Bottom: M for $t \gtrsim 0.75\mu s$ follows a different scaling with the time axis scaled according to $h_x^2 t$ (inset showing the unscaled data). The curves now collapse onto a single curve according to the h_x^2 scaling law, suggesting that the effective Hamiltonian governing the dynamics is proportional to h_x^2.

Sec. V, the h_x scaling law is expected from the effective Hamiltonian describing the coherent dynamics of a closed system at the 1-bubble resonance. On the other hand, the observed h_x^2 scaling law is a consequence of thermalization combined with a relatively slow quantum simulation measurement ramp that is performed to obtain each time point shown in the figures. The large degree of coherence in the dynamics observed in the quantum simulation is also substantiated by measurements of various bubble densities, which show that predominantly 1-bubbles are involved in changes of $M(t)$ at first, followed by other bubble sizes. Furthermore, the quality of the Rydberg blockade was also measured, substantiating the same conclusion.

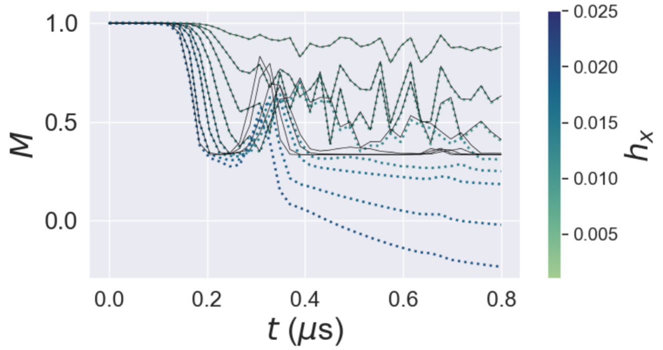

Supplementary Figure 15. Bloch-Redfield numerical emulation of magnetization M in the case of the 1-bubble resonance at $h_z = -2J$ for different values of h_x shown on the color bar on the right. The black curve shows the prediction of the effective model discussed in Sec. V, which initially overlaps with the full model and then begins to diverge. These results should be compared with the annealer data in Supplementary Figure 13.

VII. NUMERICAL EMULATION OF THE $h_z = -2J$ RESONANCE

Supplementary Figure 15 shows our Bloch-Redfield emulation results for the 1-bubble resonance at $h_z = -2J$. We emulate the magnetization M in the full model [Eq. (1)] and compare it with the magnetization in the effective model derived in Eq. (11). The effective model only considers states with the same classical energy at the given resonance, thus it provides a more tractable description of the relevant dynamical processes. At the same time, given that the effective model is restricted to a smaller corner of the Hilbert space from which it cannot escape, it will generally start to deviate from the full model after some time.

Due to the computationally demanding nature of the Bloch-Redfield emulation that requires evolving the full density matrix, in Supplementary Figure 15 we use a small system size of 3 spins. However, even such a small system size is sufficient to qualitatively reproduce several of the observed features in the 5564-spin quantum simulations in Supplementary Figure 13. For example, there is a clear modulation of M after the initial drop that coincides directly with the modulation in h_z. There is also a clear dependence of the rate of dynamics on h_x, which persists even in the presence of added decay. The effective model overlaps with the full model during initial time evolution, driven by $h_z(t)$, and then increasingly deviates from the full model dynamics.

Furthermore, by analogy with Supplementary Figure 14, we can extract two characteristic timescales that appear in the emulated dynamics of magnetization by rescaling the time axis with h_x or h_x^2. This analysis is presented in Supplementary Figure 16. We observe that the coherent scaling law predicted by theory is respected during the initial time evolution: if we scale the time axis

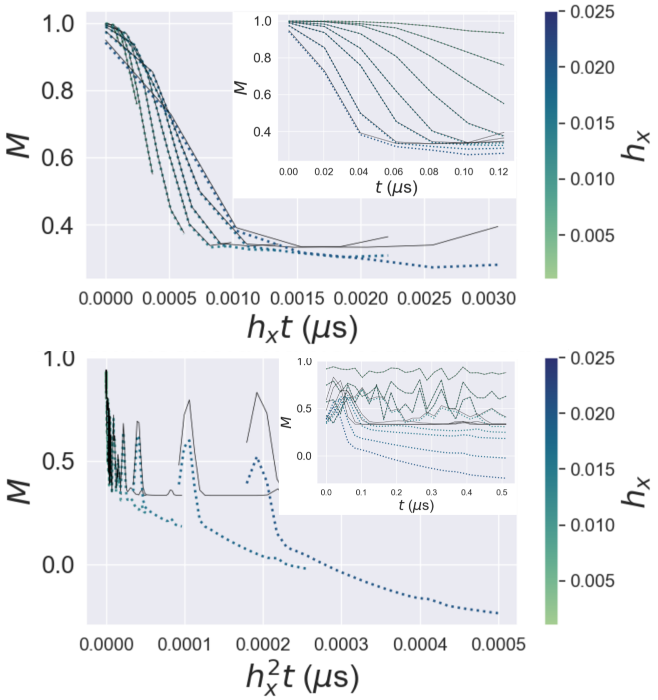

Supplementary Figure 16. Different timescales in the Bloch-Redfield numerical emulation of magnetization M in the case of 1-bubble resonance at $h_z = -2J$ from Supplementary Figure 15. Top: Short-time dynamics of M plotted as function of scaled time axis $h_x t$ for different values of h_x shown on the color bar, with the raw data shown in the inset. The curves collapse onto a single curve according to the h_x scaling law, suggesting that the effective Hamiltonian governing the dynamics is proportional to h_x. The black curves show the magnetization in the effective model derived in Eq. (11). Bottom: Magnetization at later times plotted as a function of scaled time axis $h_x^2 t$. The curves collapse onto a single curve according to the h_x^2 scaling law, suggesting that the effective Hamiltonian governing the dynamics is proportional to h_x^2. The black curves show the effective model magnetization. These results should be compared with the annealer data in Supplementary Figure 14.

according to $h_x t$, we observe a collapse of M curves, see the top panel in Supplementary Figure 16. This scaling subsequently transitions to the next order $h_x^2 t$, as can be seen in the bottom panel of Supplementary Figure 16. This suggests that the initial time evolution is driven by the external longitudinal field $h_z(t)$ and is the same as coherent quantum evolution. Dynamics only occur near the resonance after $h_z(t)$ has flipped its sign and closed in on its final value. Due to low pass filtering effects implemented on the device's hardware, which are primarily there to suppress noise influence on the qubits through the control lines, $h_z(t)$ does not monotonously reach its final value, but oscillates a few times around it and simultaneously drives M dynamics. After it settles at approximately $0.75\mu s$, thermalization effects take over, meaning we can observe coherent scaling laws up to $1\mu s$ if we include measurement ramp duration. Coherent emulation

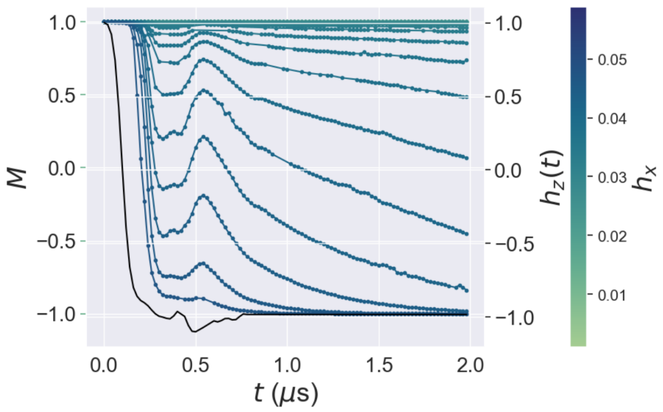

Supplementary Figure 17. Measurements of magnetization M at the 2-bubble resonance $h_z = -J$ for different values of h_x shown on the color bar. The black curve shows the $h_z(t)$ measured directly on the device with its scale on the right axis of the plot.

of larger systems, presented in the main text, shows that only 1-bubbles participate in the dynamics, which is also the case in the effective model, further supporting the h_x scaling law observed in quantum simulations.

The fate of the system after $h_z(t)$ settles is hard to conclude from our emulation results due to system size limitations. What is clear from n-bubble density measurements during Bloch-Redfield emulation is that bubbles other than 1-bubbles appear in the system. We also know from analytical considerations (see Sec. V) that 1-bubbles can move easily and collide, triggering complex interaction effects. For system sizes of 3 and 4 spins, we can reproduce the h_x^2 scaling law with or without the measurement ramp, which suggests that such behavior is ubiquitous in the 1-bubble resonance at $h_z = -2J$ after $h_z(t)$ settles. However, larger system sizes are necessary to obtain conclusive results.

VIII. QUANTUM SIMULATION OF THE $h_z = -J$ RESONANCE

In contrast to 1-bubble resonance discussed in Sec. VI above, at the 2-bubble resonance $h_z = -J$ we anticipate some differences due to the effective Hamiltonian being proportional to h_x^2 [Eq. (14)]. Supplementary Figure 17 shows measurements of the 2-bubble resonance on the quantum annealer using the protocol presented in the main text. Up to approximately $0.75\mu s$, $M(t)$ curves change according to the modulation of $h_z(t)$, with a varying degree of added decay that depends on h_x. Increasing h_x increases the rate of M decay towards the value -1, which is consistent with simply increasing the rate of dynamics. After the modulation of $h_z(t)$ settles, dynamics change qualitatively, showing only an h_x dependent decay towards the value -1, which represents the ground state of the system. To observe dynamics on

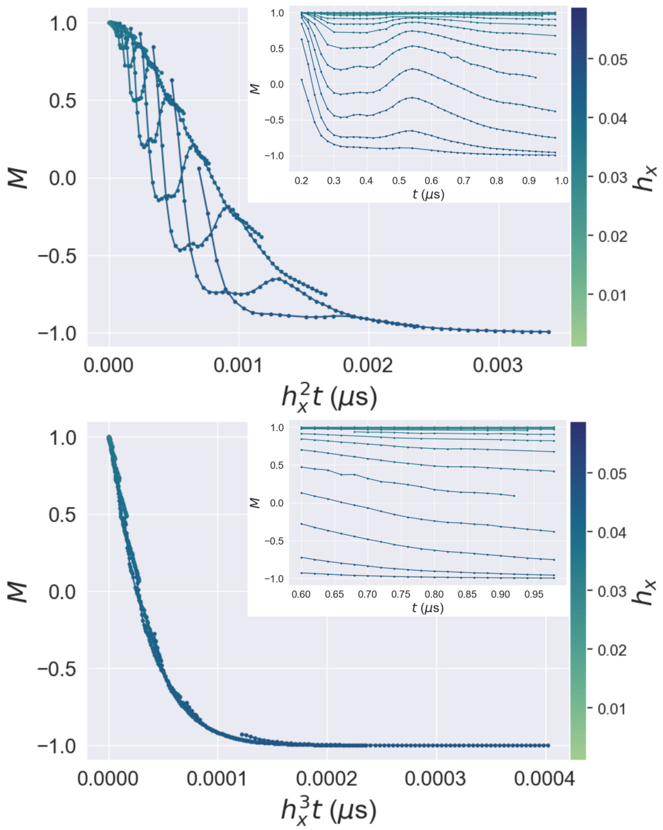

Supplementary Figure 18. Different timescales in the dynamics of magnetization M measured at the 2-bubble resonance $h_z = -J$ in Supplementary Figure 17. Top: The short-time dynamics of M with a scaled time axis $h_x^2 t$ for different values of h_x shown on the color bar, with the raw data shown in the inset. The curves collapse onto a single curve according to the h_x^2 scaling law, suggesting that the leading-order effective Hamiltonian governing the dynamics at the 2-bubble resonance is proportional to h_x^2. Bottom: The later-time dynamics of M collapse when the time axis is scaled according to $h_x^3 t$, suggesting that the effective Hamiltonian governing the dynamics is proportional to h_x^3.

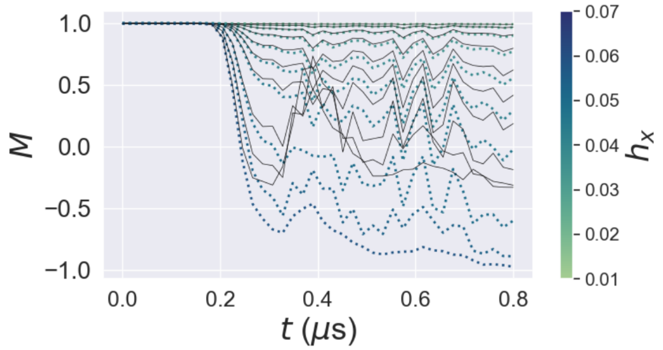

Supplementary Figure 19. Bloch-Redfield numerical emulation of magnetization M in the case of the $h_z = -J$ resonance at different times t for different values of h_x shown on the color bar on the right. The black curves show the effective model magnetization, which initially overlaps with the full model and then begins to diverge. The results should be compared with the annealer data in Supplementary Figure 17.

the time scales available on the quantum annealer, we have to increase h_x by approximately an order of magnitude, otherwise M does not change. This is consistent with theoretical predictions which state that the effective Hamiltonian scales as h_x^2 (slower), compared to h_x in the 1-bubble resonance at $h_z = -2J$.

Supplementary Figure 18 identifies the relevant timescales in the dynamics of $M(t)$ by collapsing parts of the $M(t)$ curves as the time axis is scaled with either h_x^2 or h_x^3. Below we will show, via our numerical emulation of the quantum annealer, that h_x^2 scaling law is the same as in the case of coherent or closed system quantum dynamics, while h_x^3 scaling is a consequence of thermalization combined with a relatively slow quantum simulation measurement ramp that is performed to obtain each time point shown in the figures.

IX. NUMERICAL EMULATION OF THE $h_z = -J$ RESONANCE

Supplementary Figure 19 shows our Bloch-Redfield emulation results for the 2-bubble resonance at $h_z = -J$. Similar to the 1-bubble resonance at $h_z = -2J$, discussed in Sec. VII, we emulate the magnetization in the full model and compare it with the prediction of the effective model, which is now given by Eq. (14). Moreover, similar to the 1-bubble resonance, due to the numerical complexity of the Bloch-Redfield emulation, we are restricted to small system sizes of 3 spins.

Our Bloch-Redfield results in Supplementary Figure 19 reproduce many of the observed features in the 5564-spin quantum simulations on the quantum annealer in Supplementary Figure 17. In particular, there is a clear modulation of magnetization after the initial drop that coincides directly with the modulation in h_z. There is also a clear dependence of the rate of dynamics on h_x, which persists even in the presence of added decay. The effective model overlaps with the full model during initial time evolution, driven by $h_z(t)$ and then begins to increasingly deviate from the full model dynamics. As discussed in Sec. VII, this is generally expected from the effective model that only captures a subset of all dynamical processes.

Furthermore, similar to the approach used for the 1-bubble resonance in Sec. VII, we can elucidate different timescales that play a role in the dynamics of magnetization, see Supplementary Figure 20. We observe that the coherent scaling law predicted by theory is respected during the initial time evolution: if we scale the time axis with h_x^2, different M curves collapse. At later times, the collapse is obtained by scaling with h_x^3, see bottom panel of Supplementary Figure 20. This suggests that the initial time evolution is driven by the external longitudinal field $h_z(t)$ and is the same as coherent quantum evolution. Dynamics only occur near the resonance af-

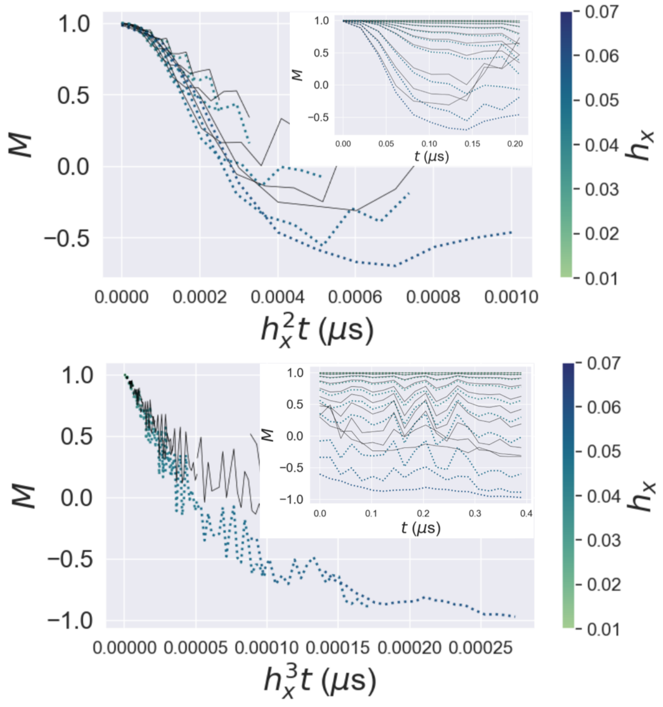

Supplementary Figure 20. Different timescales in the Bloch-Redfield numerical emulation of magnetization M in the case of 2-bubble resonance at $h_z = -J$ resonance from Supplementary Figure 19. Top: Short-time dynamics of M plotted as a function of scaled time axis $h_x^2 t$ for different values of h_x shown on the color bar, with the raw data shown in the inset. The curves collapse onto a single curve according to the h_x^2 scaling law, suggesting that the effective Hamiltonian governing the dynamics is proportional to h_x^2. The black curves show the magnetization in the effective model derived in Sec. V. The results should be compared with the annealer data in Supplementary Figure 18.

ter $h_z(t)$ has flipped its sign and closed in on its final value. Due to low pass filtering effects implemented on the device's hardware, which are primarily there to suppress noise influence on the qubits through the control lines, $h_z(t)$ does not monotonously reach its final value, but oscillates a few times around it and simultaneously drives M dynamics. After it settles at approximately $0.75\mu s$, thermalization effects take over, meaning we can observe coherent scaling laws up to $1\mu s$ if we include measurement ramp duration. Coherent emulations of the full model, presented in the main text, show that predominantly 2-bubbles participate in the dynamics, which is also the case in the effective model, thereby explaining the h_x^2 scaling law observed in quantum simulations.

The behavior of the system after $h_z(t)$ settles is hard to ascertain from our emulation results due to system-size limitations. However, the n-bubble density measurements during the Bloch-Redfield emulation show that bubbles other than 2-bubbles appear in the system. We also know from analytical considerations (Sec. V) that 2-bubbles cannot move and collide, meaning that interaction effects between bubbles can occur only after enough bubbles have been created. The h_x^3 scaling law can only be reproduced when we include the influence of the measurement ramp, unlike in the 1-bubble case. During the Bloch-Redfield numerical emulation of thermalization effects, we observe an h_x^2 scaling law for system sizes 3 and 4 and an h_x scaling law in the case of 5 spins. Larger system sizes are therefore necessary to obtain conclusive results.

---

[1] D-Wave Systems, *Technical Description of the D-Wave Quantum Processing Unit*, Tech. Rep. (D-Wave Systems Inc., Burnaby, BC, Canada, 2020) D-Wave User Manual 09-1109A-V.

[2] M. Suzuki, Generalized Trotter's formula and systematic approximants of exponential operators and inner derivations with applications to many-body problems, Commun. Math. Phys. **51**, 83 (1976).

[3] M. Suzuki, Decomposition formulas of exponential operators and Lie exponentials with some applications to quantum mechanics and statistical physics, J. Math. Phys. **26**, 601 (1985).

[4] H. F. Trotter, On the product of semi-groups of operators, Proc. Amer. Math. Soc. **10**, 545 (1959).

[5] H. De Raedt, Product formula algorithms for solving the time dependent Schrödinger equation, Comp. Phys. Rep. **7**, 1 (1987).

[6] J. Huyghebaert and H. De Raedt, Product formula methods for time-dependent Schrödinger problems, J. Phys. A: Math. Gen. **23**, 5777 (1990).

[7] K. De Raedt, K. Michielsen, H. De Raedt, B. Trieu, G. Arnold, M. Richter, T. Lippert, H. Watanabe, and N. Ito, Massively parallel quantum computer simulator, Comput. Phys. Commun. **176**, 121 (2007).

[8] H. De Raedt, F. Jin, D. Willsch, M. Willsch, N. Yoshioka, N. Ito, S. Yuan, and K. Michielsen, Massively parallel quantum computer simulator, eleven years later, Comput. Phys. Commun. **237**, 47 (2019).

[9] D. Willsch, M. Willsch, F. Jin, K. Michielsen, and H. De Raedt, GPU-accelerated simulations of quantum annealing and the quantum approximate optimization algorithm, Comput. Phys. Commun. **278**, 108411 (2022).

[10] S. Bravyi, D. P. DiVincenzo, and D. Loss, Schrieffer-wolff transformation for quantum many-body systems, Annals of Physics **326**, 2793 (2011).

[11] A. Sinha, T. Chanda, and J. Dziarmaga, Nonadiabatic dynamics across a first-order quantum phase transition: Quantized bubble nucleation, Phys. Rev. B **103**, L220302 (2021).

[12] P. Fendley, K. Sengupta, and S. Sachdev, Competing density-wave orders in a one-dimensional hard-boson model, Phys. Rev. B **69**, 075106 (2004).

[13] I. Lesanovsky and H. Katsura, Interacting Fibonacci anyons in a Rydberg gas, Phys. Rev. A **86**, 041601(R) (2012).

[14] H. Bernien, S. Schwartz, A. Keesling, H. Levine, A. Omran, H. Pichler, S. Choi, A. S. Zibrov, M. Endres, M. Greiner, V. Vuletić, and M. D. Lukin, Probing many-body dynamics on a 51-atom quantum simulator, Nature **551**, 579 (2017).

[15] C. J. Turner, A. A. Michailidis, D. A. Abanin, M. Serbyn, and Z. Papić, Weak ergodicity breaking from quantum many-body scars, Nat. Phys. **14**, 745 (2018).

[16] G.-X. Su, H. Sun, A. Hudomal, J.-Y. Desaules, Z.-Y. Zhou, B. Yang, J. C. Halimeh, Z.-S. Yuan, Z. Papić, and J.-W. Pan, Observation of many-body scarring in a Bose-Hubbard quantum simulator, Phys. Rev. Res. **5**, 023010 (2023).

[17] D. Bluvstein, A. Omran, H. Levine, A. Keesling, G. Semeghini, S. Ebadi, T. T. Wang, A. A. Michailidis, N. Maskara, W. W. Ho, S. Choi, M. Serbyn, M. Greiner, V. Vuletić, and M. D. Lukin, Controlling quantum many-body dynamics in driven Rydberg atom arrays, Science **371**, 1355 (2021).
